# Naturalizing relevance realization: why agency and cognition are fundamentally not computational

**DOI:** 10.3389/fpsyg.2024.1362658

**Published:** 2024-06-25

**Authors:** Johannes Jaeger, Anna Riedl, Alex Djedovic, John Vervaeke, Denis Walsh

**Affiliations:** ^1^Department of Philosophy, University of Vienna, Vienna, Austria; ^2^Complexity Science Hub (CSH) Vienna, Vienna, Austria; ^3^Ronin Institute, Essex, NJ, United States; ^4^Middle European Interdisciplinary Master’s Program in Cognitive Science, University of Vienna, Vienna, Austria; ^5^Cognitive Science Program, University of Toronto, Toronto, ON, Canada; ^6^Institute for the History and Philosophy of Science and Technology, University of Toronto, Toronto, ON, Canada; ^7^Department of Psychology, University of Toronto, Toronto, ON, Canada; ^8^Department of Philosophy, University of Toronto, Toronto, ON, Canada

**Keywords:** relevance realization, open-ended evolution, radical emergence, autopoiesis, anticipation, adaptation, natural agency, cognition

## Abstract

The way organismic agents come to know the world, and the way algorithms solve problems, are fundamentally different. The most sensible course of action for an organism does not simply follow from logical rules of inference. Before it can even use such rules, the organism must tackle the problem of relevance. It must turn ill-defined problems into well-defined ones, turn semantics into syntax. This ability to realize relevance is present in all organisms, from bacteria to humans. It lies at the root of organismic agency, cognition, and consciousness, arising from the particular autopoietic, anticipatory, and adaptive organization of living beings. In this article, we show that the process of relevance realization is beyond formalization. It cannot be captured completely by algorithmic approaches. This implies that organismic agency (and hence cognition as well as consciousness) are at heart *not* computational in nature. Instead, we show how the process of relevance is realized by an adaptive and emergent triadic dialectic (a trialectic), which manifests as a metabolic and ecological-evolutionary co-constructive dynamic. This results in a meliorative process that enables an agent to continuously keep a grip on its arena, its reality. To be alive means to make sense of one’s world. This kind of embodied ecological rationality is a fundamental aspect of life, and a key characteristic that sets it apart from non-living matter.

*“To live is to know.”*

(Maturana, 1988)

*“Between the stimulus and the response, there is a space. And in that space lies our freedom and power to choose our responses.”*

(Frankl, 1946, 2020)

*“Voluntary actions thus demonstrate a ‘freedom from immediacy.’ ”*

(Haggard, 2008; channeling Shadlen and Gold, 2004)

## 1 Introduction

All organisms are limited beings that live in a world overflowing with potential meaning (Varela et al., 1991; Weber and Varela, 2002; Thompson, 2007), a world profoundly exceeding their grasp (Stanford, 2010). Environmental cues likely to be important in a given situation tend to be scarce, ambiguous, and fragmentary. Clear and obvious signals are rare (Felin and Felin, 2019).^1^ Few problems we encounter in such a “large world” are well-defined (Savage, 1954). On top of this, organisms constantly encounter situations they have never come across before. To make sense of such an ill-defined and open-ended world—in order to survive, thrive, and evolve—the organism must first realize what is relevant in its environment. It needs to solve *the problem of relevance*.

In contrast, algorithms—broadly defined as automated computational procedures, i.e., finite sets of symbols encoding operations that can be executed on a universal Turing machine—exist in a “small world” (Savage, 1954). They do so by definition, since they are embedded and implemented within a predefined formalized ontology (intuitively: their “digital environment” or “computational architecture”), where all problems are well-defined. They can only mimic (emulate, or simulate) partial aspects of a large world: algorithms cannot identify or solve problems that are not precoded (explicitly or implicitly) by the rules that characterize their small world (Cantwell Smith, 2019). In such a world, everything and nothing is relevant at the same time.

This is why the way organisms come to know their world fundamentally differs from algorithmic problem solving or optimization (Roli et al., 2022; see also Weber and Varela, 2002; Di Paolo, 2005). This elementary insight has profound consequences for research into artificial intelligence (Jaeger, 2024a), but also for our understanding of natural agency, cognition, and ultimately also consciousness, which will be the focus of this article. An organism’s actions and behavior are founded on the ability to cope with unexpected situations, with cues that are uncertain, ambivalent, or outright misleading (see Wrathall and Malpas, 2000; Ratcliffe, 2012; Wheeler, 2012, for discussion). It does so by being directly embodied and embedded in its world, which allows it to actively explore its surroundings through action and perception (see, for example, Varela et al., 1991; Thompson, 2007; Soto et al., 2016a; Di Paolo et al., 2017). Algorithms do not have this ability—they cannot truly improvise, but only mimic exploratory behavior, emulate true agency—due to their rigidly formalized nature.

Contrary to an algorithm, the most sensible (and thus “rational”) course of action for an organism does not simply follow from logical rules of inference, not even abductive inference to the best explanation (see, for example, Feyerabend, 1975; Arthur, 1994; Thompson, 2007; Gigerenzer, 2021; Riedl and Vervaeke, 2022; Roli et al., 2022). Before they can “infer” anything, living beings must first turn ill-defined problems into well-defined ones, transform large worlds into small, translate intangible semantics into formalized syntax (defined as the rule-based processing of symbols free of contingent, vague, and ambiguous external referents). And they must do this incessantly: it is a defining feature of their mode of existence.

This process is called *relevance realization* (Vervaeke et al., 2012; Vervaeke and Ferraro, 2013a,b). The ability to solve the problem of relevance is a necessary condition and the defining criterion for making sense of a large world.^2^ Indeed, we could say that an organism actively *brings forth* a whole world of meaning (Varela et al., 1991; Rolla and Figueiredo, 2023).

In this article, we shall argue that the ability to realize relevance—to bring forth a world—is present in all organisms, from the simplest bacteria to the most sophisticated human beings. In fact, it is a universal feature of *any* limited living being that must make sense of its large world. Therefore, relevance realization is one of the key properties that sets apart living systems from non-living ones, such as algorithms and their concrete physical implementations, which we will call machines. The ability to solve the problem of relevance arises from the characteristic self-referential self-manufacturing dynamic organization of living matter, which enables organisms to attain a degree of self-determination, to act with some autonomy, and to anticipate the consequences that may follow from their actions.

All of this involves a radically context-dependent generative dialectic^3^ called *opponent processing*—the continual establishment of trade-offs and synergies between competing and complementary organismic behaviors and dynamics (Vervaeke et al., 2012). Such competing and synergizing processes also mediate an organism’s interactions with its living and non-living surroundings, interactions that are inherently and irreducibly semantic, in the sense of having value (i.e., relevance) for the organism as a unity which strives to persist in the face of the constant threat of decay. This manifests as mutually co-creating (and thus collectively co-emergent) interrelations between a living agent’s intrinsic goals, its repertoire of actions, and the affordances that arise from the interactions of the organism with its experienced environment (Walsh, 2015).

As we shall see, *this dialectic and hierarchical tangle of processes forms the generative core of the phenomenon of natural agency*, and therefore also of its evolutionary elaborations: cognition and consciousness (cf. Felin and Koenderink, 2022). There is nothing mystifying or obscurantist about this view. In fact, we can demonstrate that this dialectic is straightforwardly analogous to the dynamics of an evolving population of biological individuals, where different survival strategies are played out against each other to bring about adaptation through natural selection (Vervaeke et al., 2012). It is, in essence, a Darwinian adaptive evolutionary dynamic. Our central claim in what follows is that *this dialectic dynamic of relevance realization is not an algorithmic process, due to its fundamentally impredicative and co-constructive nature.* Therefore, *natural agency, cognition, and consciousness are, at their very core, not computational phenomena*, and if we restrict ourselves to study them by purely computational means, we are likely to miss the point entirely.

We will carefully unpack this rather dense and compact mission statement in the following sections. As our starting point, let us take the analogy of relevance and evolutionary fitness implied above (see also Vervaeke et al., 2012).^4^ Both concepts are closely related in the sense of pertaining to a radically context-dependent match between an agent and its immediate task-relevant environment, or *arena* (Vervaeke et al., 2017). Neither “fitness” nor “relevance” have any universal attributes: there is no trait that renders you fit in all environments, nor is there any factor that is relevant across all possible situations. Furthermore, both fitness and relevance can only be assessed in a relative manner: one individual in a specific population and environment is fitter than another, and some features of the world may be more relevant than others. In other words, these concepts are only explanatory when used in an appropriate comparative context.

Because of this close conceptual analogy between relevance and fitness, *an understanding of relevance realization ought to be of an evolutionary, ecological, and economic nature*, formulated as the interplay of competing adaptive processes, situated in a particular setting, and phrased in terms of the variable and weighed commitments of organisms to a range of different goals.

Opponent processing means that organisms constantly play different approaches and strategies against each other in a process that iteratively evaluates progress toward a problem solution (toward reaching a particular goal). This can include dynamically switching back and forth between opposing strategies, if the necessity arises. By identifying molecular, cellular, organismic, ecological, and evolutionary processes and relations that can implement such an adaptive dynamic, we can generalize the concept of relevance realization from the fundamental prerequisite for cognition in animals with nervous systems (including us humans, of course, Vervaeke et al., 2012) to the fundamental prerequisite underlying natural agency in *all* living organisms.

It is important to mention at the outset that our project—to naturalize relevance realization—happens in an incredibly rich historical context of established research traditions. It is not our aim here to provide a careful review of all of those traditions (although we will do our best to refer to them wherever relevant). Instead, we see relevance realization as a promising framework that could provide a conceptual bridge between empirical approaches to organismic biology and the cognitive neurosciences, research into artificial intelligence, and intellectual traditions such as evolutionary epistemology (see, for example, Campbell, 1974; Lorenz, 1977; Bradie, 1986; Cziko, 1995; Bradie and Harms, 2023; but also Wimsatt, 2007), ecological psychology (Gibson, 1966, 1979), 4E cognition (Di Paolo et al., 2017; Durt et al., 2017; Newen et al., 2018; Carney, 2020; Shapiro and Spaulding, 2021), and biosemiotics (Favareau, 2010; Deacon, 2011, 2015; Pattee and Rączaszek-Leonardi, 2012; Barbieri, 2015). We draw from all of these in our argument. Note, however, that to achieve our aim of cross-disciplinary translation, we will deliberately try to avoid the specific terminologies of these various domains, and express ourselves in terms that we hope are easily accessible to a wide range of researchers across relevant disciplines.

This article is structured as follows: section “2 Agential emergentism” starts by outlining a number of problems we encounter when considering cognition (and the world) as some form of algorithmic computation and introduces our proposed alternative perspective. In section “3 Relevance realization,” we characterize the process of relevance realization, outlining its foundational role in cognition and in the choice of rational strategies to solve problems. Section “4 Biological organization and natural agency” introduces the notion of biological organization, shows how it emerged as a novel kind of organization of matter at the origin of life, and demonstrates how it enables basic autonomy and natural agency through the organism’s ability to set its own intrinsic goals. The pursuit of its goals requires an organism to anticipate the consequences of its actions. How this is achieved even in the simplest of creatures is the topic of section “5 Basic biological anticipation.” In section “6 Affordance, goal, and action,” we examine the adaptive dialectic (or trialectic) interplay between an organism’s goals, actions, and affordances. As the core of our argument, we bring this model to bear on the process of relevance realization itself, and show how this process cannot be captured completely by any kind of algorithm. In section “7 To live is to know,” we synthesize the insights from the previous three sections, to show how the evolution of cognition in organisms with a nervous system, and perhaps also consciousness, can be explained as adaptations to realizing relevance in ever more complex situations. In section “8 Conclusion,” we conclude our argument by applying the concept of relevance realization to rational strategies for problem solving. We argue that a basic ecological notion of rational behavior is common to all living beings. In other words, organisms make sense of the world in a way that makes sense to them.

## 2 Agential emergentism

Contemporary approaches to *natural agency* and *cognition* take a very wide range of stances on what these two concepts mean, what they refer to, and how they relate to each other. Some of these approaches consider cognition a fundamental property of all living beings (see, for example, Maturana and Varela, 1980; Varela et al., 1991; Lyon, 2006; Baker and Stock, 2007; Shapiro, 2007; Thompson, 2007; Baluška and Levin, 2016; Birch et al., 2020; Lyon et al., 2021), some treat it as restricted to animals with sufficiently complex internal models of the world (Djedovic, 2020; Sims, 2021) or in possession of a nervous system (e.g., Moreno et al., 1997; Moreno and Etxeberria, 2005; Moreno and Mossio, 2015; Di Paolo et al., 2017). Some see natural agency as a phenomenon that is clearly distinct from cognition (see, for example, Kauffman, 2000; Barandiaran and Moreno, 2008; Rosen, 2012; Moreno and Mossio, 2015; Fulda, 2017), some consider the two to be the same (e.g., Maturana and Varela, 1980; Lyon, 2006; Thompson, 2007; Baluška and Levin, 2016). Among this diversity of perspectives, we can identify two general trends in attitudes. Let us call them *agential emergentism* and *computationalism*.^5^

Computationalism, as we use the term here, encompasses various forms of cognitivism and connectionism (cf. classification by Thompson, 2007). It is extremely popular and widespread in contemporary scientific and philosophical thinking, the basic tenet being that both natural agency and cognition are special varieties of algorithmic computation. Computationalism formulates agential and cognitive phenomena in terms of (often complicated, nonlinear, and heavily feedback-driven) input-output information processing (see, Baluška and Levin, 2016; Levin, 2021, for a particularly strong and explicit example). In this framework, goal-directedness—sometimes unironically called “machine wanting” (McShea, 2013)—tends to be explained by some kind of cybernetic homeostatic regulation (e.g., McShea, 2012, 2013, 2016; Lee and McShea, 2020).

The strongest versions of computationalism assert that all physical processes which can be actualized (not just cognitive ones) must be Turing-computable. This pancomputationalist attitude is codified in the *strong* (or *physical*) *Church-Turing conjecture* (also called *Church-Turing-Deutsch conjecture*; Deutsch, 1985, 1997; Lloyd, 2006).^6^ It is fundamentally reductionist in nature, an attempt to force the explanation of all of physical reality in terms of algorithmic computation based on lower-level mechanisms. For this reason, computation is not considered exclusive to living systems. Researchers in the pancomputationalist paradigm see agency and cognition as continuous with non-linear and self-organizing information processing outside the living world (see, for example, Levin, 2021; Bongard and Levin, 2023). On this view, there is no fundamental boundary between the realms of the living and the non-living, between biology and computer engineering. The emergence of natural agency and cognition in living systems is simply due to a (gradual) increase in computational complexity and capacity in the underlying physical processes.

We consider this view of reality to be highly problematic. One issue with pancomputationalism is that physical processes and phenomena are generally not discrete by nature (at least not obviously so) and are rarely deterministic in the sense that algorithmic processes are (Longo, 2009; Longo and Paul, 2011). We will not dwell on this here, but will focus instead on the relation between syntactic formal systems and a fundamentally semantic and ill-defined large world. In this context, it is crucial to distinguish the ability to algorithmically simulate (i.e., approximate) physical and cognitive processes from the claim that these processes intrinsically *are* a form of computation. The latter view mistakes the map for the territory by misunderstanding the original purpose of the theory of computation: as defined by Church and Turing, “computation” is a rote procedure performed by a human agent (the original “computer”) carrying out some calculation, logical inference, or planning procedure (Church, 1936; Turing, 1937; see, Copeland, 2020, for an historical review). The theory of computation was intended as a model of specific human activities, not a model of the brain or physical reality in general. Consequently, assuming that the brain or the world in general *is* a computer means committing a category mistake called *the equivalence fallacy* (Copeland, 2020). Treating the world as computation imputes symbolic (information) content onto physical processes that is only really present in our simulations, not in the physical processes that we model. The world we directly experience as living organisms is not formalized and, in fact, is not formalizable completely by any limited being, as we shall see in section “3. Relevance realization.” This poses an obvious and fundamental problem for the pancomputationalist view.

To better understand and ultimately overcome this problem, we adopt an alternative stance called *agential emergentism* (outlined in detail in Walsh, 2013, 2015). The basic idea is to provide a fresh and expanded perspective on life that allows us to bridge the gap between the syntactic and the semantic realms, between small and large worlds. Agential emergentism postulates that all organisms possess a kind of *natural agency*. Note that this is not the same as the so-called *intentional stance* (Dennett, 1987; recently reviewed in Okasha, 2018), which merely encourages us to treat living systems *as if* they had agency while retaining a thoroughly reductionist worldview (see also the teleonomy account of Mayr, 1974, 1982, 1992). In contrast, agential emergentism treats agency as natural and fundamental: the key property that distinguishes living from non-living systems (Moreno and Mossio, 2015; Walsh, 2015; Mitchell, 2023). Only organisms—not algorithms or other machines—are *true agents*, because only they can act on their own behalf, for their own reasons, in pursuit of their own goals (Kauffman, 2000; Nicholson, 2013; Walsh, 2015; Roli et al., 2022; Mitchell, 2023; Jaeger, 2024a).

We can define *natural agency* in its broadest sense as the capability of a living system to initiate actions according to its own internal norms (Di Paolo, 2005; Barandiaran et al., 2009; Moreno and Mossio, 2015; Djedovic, 2020; Walsh and Rupik, 2023). This capability arises from the peculiar self-referential and hierarchical causal regime that underlies the self-manufacturing organization of living matter (see section “4 Biological organization and natural agency”). A lower bound for normativity arises from such self-manufacture: the right thing to do is what keeps me alive (Weber and Varela, 2002; Di Paolo, 2005; Di Paolo et al., 2017; Djedovic, 2020). This imposes a discrete discontinuity between the realms of the living and the non-living: algorithms and machines only possess extrinsic purpose, imposed on them from outside their own organization, while organismic agents can, and indeed must, define their own intrinsic goals (see also Nicholson, 2013; Mossio and Bich, 2017).

This is why it makes good sense to treat relevance realization from an agential rather than computationalist perspective: the ability to solve the problem of relevance is intimately connected to the possession of intrinsic goals. To put it simply: if you do not truly want or desire anything, if there is nothing that is good or bad in your world, you cannot realize what is relevant for you. Phrased a bit less informally: the relevance of certain features of a situation to the organism is a function of the organism’s goals and how the situation promotes or impedes them. This is why algorithms in their small worlds cannot solve the problem of relevance: they never even encounter it! In addition, relevance realization requires an organism to assess potential outcomes of its behavior.^7^ To realize what is relevant in a given situation, you have to be able to somehow anticipate the consequences of your actions. On top of all this, an organism must be motivated to pursue its goals. Motivation ultimately stems from our fragility and mortality (Jonas, 1966; Weber and Varela, 2002; Thompson, 2007; Deacon, 2011; Moreno and Mossio, 2015). While it is possible to impose external motivation on a system that mimics aspects of internal motivation, true internal motivation can only arise from precariousness. We must be driven to continue living. Without this drive, we do not get the proper Darwinian dynamics of open-ended evolution (as argued in detail in Roli et al., 2022; Jaeger, 2024b). In what follows, we develop a naturalistic evolutionary account of relevance realization that is framed based on this simple set of basic principles.

## 3 Relevance realization

Organisms actively explore their world through their actions. For an organismic agent, selecting an appropriate action in a given situation poses a truly formidable challenge. How do living systems—including us humans—even begin to tackle the problems they encounter in their environment, considering that they live in a large world that contains an indefinite (and potentially infinite) number of features that may be relevant to the situation at hand? To address this question, we must first clarify what kind of environment we are dealing with.

It is not simply the external physical environment that matters to the organism, but its *experienced environment*, the environment it perceives, the environment that makes a difference for choosing how to act (sometimes called the organism’s *umwelt*; von Uexküll, 1909; Thompson, 2007; Walsh D. M., 2012). Both paramecia and porpoises, for example, live in the physical substance “water,” but due to their enormous size difference, they have to deal with very distinct sensorimotor contexts concerning their propulsion through that physical medium (Walsh, 2015). What matters most on the minuscule scale of the paramecium is the *viscosity* of water. It “digs” or “drills” its way through a very syrupy medium. The porpoise, in contrast, needs to solve problems of *hydrodynamics* at a much larger scale. It experiences almost none of the viscosity but all the fluidity of water, hence the convergent evolution of fish-like body shape and structures like flippers that enhance its hydrodynamic properties.

This simple example illustrates an important general point: what is relevant to an organism in its environment is never an entirely subjective or objective feature. Instead, it is *transjective*, arising through the interaction of the agent with the world (Vervaeke and Mastropietro, 2021a,b). In other words, the organism *enacts*, and thereby brings forth, its own world of meaning and value (Varela et al., 1991; Weber and Varela, 2002; Di Paolo, 2005; Thompson, 2007; Rolla and Figueiredo, 2023). This grounds the process of *relevance realization* in a constantly changing and evolving *agent-arena relationship*, where “arena” designates the situated and task-relevant portion of the larger experienced environment (see Vervaeke et al., 2017, p. 104). The question of relevance then becomes the question of *how an agent manages to delimit the appropriate arena*, to single out the task-relevant features of its experienced environment, given its specific situation.

For the computationalist, singling out task-relevant features is indistinguishable from problem solving itself, and both must be subsumed under an algorithmic frame. If we take the human context of scientific inquiry as an example, inductive, deductive, and abductive inference—all adhering to explicitly formalized logical rules—are generally deemed sufficient for identifying, characterizing, and solving research problems (see, Roli et al., 2022, for a critical discussion). Accordingly, the *general problem solving framework* by Newell and Simon (1972) delimits a problem formally by requiring specific initial and goal states, plus a set of operators (actions) used by the problem-solving agent to transition from the former to the latter within a given set of constraints. A problem solution is then defined as a sequence of actions that succeeds in getting the agent from the initial state to the attainment of its goal. The agent’s task is prescribed as solving a formal optimization problem that identifies solutions to a given challenge. To a computationalist, the foundation of natural agency and cognition is formal problem solving.

This kind of computational framing can be very useful. For instance, it points our attention to the issue of *combinatorial explosion*, revealing that for all but the most trivial problems, the space of potential solutions is truly astronomical (Newell and Simon, 1972). For problem solving to be tractable under real-world constraints, agents must rely on *heuristics*, make-shift solutions that are far from perfect (Simon and Newell, 1958). Unlike algorithms (strictly defined), they are not guaranteed to converge toward a correct solution of a well-posed problem in finite time. Still, heuristics are tried and tested to work well enough (to *satisfice*) in a range of situations which the agent or its ancestors have encountered in the past, or which the agent deems in some way analogous to such past experiences (Simon, 1956, 1957, 1989; [Bibr B174][Bibr B68]; Gigerenzer, 2021).

**FIGURE 1:**
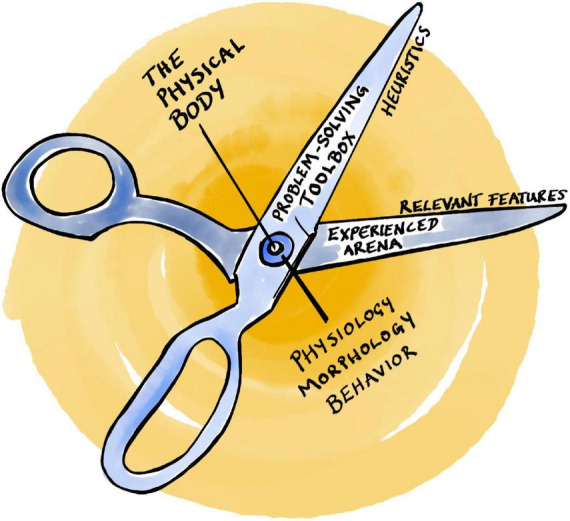
Simon’s scissors.

This notion of *bounded rationality* (Simon, 1957) is illustrated by the visual metaphor of *Simon’s scissors* with two blades that have to fit together (Figure 1; Simon, 1990): (1) the agent’s internal cognitive toolbox (with its particular set of heuristics and associated limitations), and (2) an experienced arena with a given structure of relevant features. Put simply, heuristics must be adapted to the task at hand, otherwise they do not work. On top of this, the physical body with its peculiar physiology, morphology, and sensorimotor abilities can be added to the metaphor as the pivot between the two scissor blades (Figure 1; Mastrogiorgio and Petracca, 2016; Gallese et al., 2020), reflecting the notion of *embodied bounded rationality* (Gallese et al., 2020; Petracca, 2021; Petracca and Grayot, 2023), or evolved *embodied heuristics* (Gigerenzer, 2021).

Evolved embodied heuristics can effectively reduce the problem of intractably large search spaces. They are indispensable tools for limited beings in large worlds, because they enable us to ignore a vast amount of information that is likely to be irrelevant (Brighton, 2018). Yet, they leave one central issue untouched: how to link the use of specific heuristics to the identification of underlying relevant cues (Felin et al., 2017; Felin and Koenderink, 2022). The problem of relevance thus persists. One reason for this is that heuristics remain confined to a small world after all. They are still algorithms (automated computational procedures) in the broader sense of the term.

This reveals a vicious circularity in the argument above (Vervaeke et al., 2012; Riedl and Vervaeke, 2022): it presupposes that an agent can turn ill-defined problems into problems that are defined precisely enough to be tractable heuristically. There must still be a well-defined goal and search space, a set of available actions, and the agent must be able to *categorize* its problems to judge whether a given situation is analogous to contexts encountered in the past (Vervaeke et al., 2012; see also Hofstadter and Sander, 2013). And here lies the crux: all of this requires the agent to distinguish relevant features in its experienced environment—to circumscribe its arena—*before* it can apply any heuristics. In other words, embodied heuristics presuppose a solution to the problem that they are meant to tackle in the first place.

Algorithmic approaches to relevance realization in a large world generally get us nowhere. A first challenge is that the search space required for formal optimization usually cannot be circumscribed precisely because the collection of large-world features that may be relevant in a given situation is *indefinite*: it cannot be prestated explicitly in the form of a mathematical set (Roli et al., 2022).^8^ Indeed, the collection of potentially relevant features may also be *infinite*, because even the limited accessible domain of an organism’s large world can be inexhaustibly fine-grained, or because there is no end to the ways in which it can be validly partitioned into relevant features (Kauffman, 1971; Wimsatt, 2007). Connected to this difficulty is the additional problem that we cannot define an abstract mathematical class of all relevant features across all imaginable situations or problems, since there is no essential general property that all of these features share (Vervaeke et al., 2012). What is relevant is radically mutable and situation-dependent. Moreover, the internal structure of the class of relevant features for any particular situation is unknown (if it has any predefined structure at all): we cannot say in advance, or derive from first principles, how such features may relate to each other, and therefore cannot simply infer one from another. Last but not least, framing the process of relevance realization as a formal optimization problem inexorably leads to an infinite regress: delimiting the search space for one problem poses a new optimization challenge at the next level (how to find the relevant search space limits and dimensions) that needs a formalized search space of its own, and so on and so forth.

Taken together, these problems constitute an insurmountable challenge for any limited being attempting to construct a universal formal theory of relevance for general problem solving in a large world. In other words, *relevance realization is not completely formalizable*. This makes sense if we consider that relevance realization *is* the act of formalization, of turning semantics into syntax, as we will outline in detail below. This is exactly how David Hilbert defined “formalization” for the purpose of his ultimately unsuccessful program to put mathematics on a complete and consistent logical foundation (Zach, 2023). Hilbert’s failure was made obvious by Gödel’s incompleteness theorems, which state that every sufficiently complicated formal system remains incomplete, because there will always be valid propositions that are true but cannot be proven within the existing formalism (reviewed in Nagel and Newman, 2001). If systems of mathematical propositions cannot be completely formalized, even in principle, is it really surprising that the large world we live in cannot either?

Once we accept that organisms live in a large world, and that this world is not fully formalizable, we must recognize that natural agency and cognition cannot be grounded wholly in formal problem solving, or any other form of algorithmic computation. Before they can attempt to solve any problems, organisms must first perform the basic task of formalizing or *framing the problem* they want to solve. Without this basic framing, it is impossible to formulate hypotheses for abductive reasoning or, more generally, to select actions that are appropriate to the situation at hand. Algorithms are unable to perform this kind of framing. By their nature, they are confined to the syntactic realm, always operating *within* some given formal frame—their predefined ontology—which must be provided and precoded by an external programmer (see also Jaeger, 2024a).^9^ This is true even if the framing provided is indirect and implicit, and may allow for a diverse range of optimization targets, as is the case in many contemporary approaches to unsupervised machine learning. Indeed, the inability of algorithms to frame problems autonomously has been widely recognized as one of the fundamental limitations of our current quest for artificial general intelligence (see, for example, McCarthy and Hayes, 1969; Dreyfus, 1979, 1992; Dennett, 1984; Cantwell Smith, 2019; Roitblat, 2020; Roli et al., 2022). Algorithms cannot, on their own, deal with the ambiguous semantics of a large world.

This has some profound and perhaps counterintuitive implications. The most notable of these is the following: if the frame problem, defined in its most general form as the problem of relevance (Dennett, 1984; Vervaeke et al., 2012; Riedl and Vervaeke, 2022), cannot be solved within an algorithmic framework, yet organisms are able to realize relevance, then *the behavior and evolution of organisms cannot be fully captured by formal models based on algorithmic frameworks*.

More specifically, it follows that *relevance realization cannot be an algorithmic process* itself: to avoid vicious circularity and infinite regress, it must be conceptualized as lying outside the realm of purely syntactic inferential computation (Roli et al., 2022; Jaeger, 2024a). As a direct corollary, it must also lie outside the domain of symbolic processing,^10^ which is embedded in the realm of syntax, and is therefore completely formalized just like algorithmic computation (Vervaeke et al., 2012). Note that this does not preclude that we can superficially *mimic* or *emulate* the process of relevance realization through algorithmic simulation or formal symbolic description. Remember that we are formulating an incompleteness argument here, which suggests that a purely algorithmic (syntactic) approach will never be able to capture the process of relevance realization *in its entirety*. To any limited being, there always remains some *semantic residue* in its large world that defies precise definition. To make sense of such a world, natural and cognitive agents cannot rely exclusively on algorithmic computation or symbolic processing.

How, then, are we to understand relevance realization if not in terms of formal problem solving? One possibility is through *an economic perspective* (Vervaeke et al., 2012; Vervaeke and Ferraro, 2013a,b), which frames the problem of relevance based on *commitment*, i.e., the dynamic allocation of resources by an agent to the pursuit of a range of potentially conflicting or competing goals. Opponent processing is seen as *a meta-heuristic approach*: the agent employs a number of complementary or even antagonistic heuristics that are played against each other in the presence of different kinds of challenges and trade-offs. The trade-offs involved can be subsumed under the general opposition of efficiency vs. resilience or, more specifically, as generality vs. specialization, exploration vs. exploitation, and focusing vs. diversifying (Vervaeke et al., 2012; Andersen et al., 2022). On this account, resource allocation is fundamentally dialectic: the agent continually reassesses what strategy does or does not work in a given situation, and adjusts its goals and priorities accordingly, which in turn affects its appraisal of progress. This leads to a situated and temporary adaptive fit between agent and arena, which is continuously updated according to the experienced environment, anticipated outcomes of actions, and the inner state of the agent. Overall, it accounts for the context-specific nature of relevance realization in terms of localized adaptive dynamics.

Such high-level adaptive dynamics can be embedded in a physical context through the notion of *predictive processing* (see, for example, Friston, 2010, 2013; Clark, 2013, 2015; Hohwy, 2014; Andrews, 2021; Colombo and Wright, 2021; Seth, 2021; Andersen et al., 2022). Predictive processing means that an agent iteratively and recursively evaluates the relevance of its sensory input through the estimation of prediction errors. It does this by measuring the discrepancy between expectations based on its internal models of the world (see section “5 Basic biological anticipation”) and the sensory feedback it receives from its interactions within its current arena. Higher weights are assigned to input with low prediction errors, while perceptions with persistent larger errors are preferentially discounted. Particular importance is attributed to *error dynamics*, the selection of actions and cognitive strategies that rapidly reduce prediction errors in a particular stream of sensory input (Friston et al., 2012; Kiverstein et al., 2019; Andersen et al., 2022). Predictive processing can ground the economic account of relevance realization by connecting it to the underlying perceptual and cognitive processes that account for the dynamic and recurrent weighing of prediction errors.

For our present purposes, however, both of these accounts exhibit several significant limitations. The economic account was developed specifically in the context of human cognition, and it is not entirely clear whether it can be generalized beyond that scope. It takes agency (even intention) for granted without providing a naturalistic justification for that assumption. The commitment of resources in the economic account, and the assignment of precision weights in predictive processing, presuppose that the agent *has* intrinsic goals in relation to which such actions can be taken (Andersen et al., 2022). This presupposition may be acceptable in the context of human cognition, with its evident and explicit intentionality, but poses a serious challenge for generalizing relevance realization to the domain of non-human and (even more so) non-cognitive living beings.

On top of all this, both the economic account and formalized versions of predictive processing remain embedded in a thoroughly computationalist framework that views the allocation of relevance (and hence resources or precision weights) as a simple iterative and recursive algorithmic process. This begs the question where organismic goals come from in the first place, and how anticipated outcomes can affect the strategies and actions chosen. For these reasons, we will take a different route, with the aim of grounding the process of relevance realization in the basic organization of living beings and the kind of agent-arena relationship this organization entails. Our approach is not intended to oppose the economic account or the account based on predictive processing, but rather to ground them in the natural sciences with the aim of extending relevance realization beyond human cognition and intentionality. It is *an evolutionary view of relevance realization*, which also provides an explanation of why it is that the process cannot be completely captured by a purely syntactic or algorithmic approach.

## 4 Biological organization and natural agency

The ability to solve the problem of relevance crucially relies on an agent setting intrinsic goals. Therefore, we first need to demonstrate that all organisms can define and pursue their own goals without requiring any explicit intentionality, cognitive capabilities, or consciousness. Such basic *natural agency* does not primarily rely on causal indeterminacy or randomness. Instead, it rests in the peculiar self-referential and hierarchical causal regime that underlies the organization of living matter (see, for example, Rosen, 1958a,b, 1959, 1972, 1991; Piaget, 1967; Varela et al., 1974; Varela, 1979; Maturana and Varela, 1980; Juarrero, 1999, 2023; Kauffman, 2000; Weber and Varela, 2002; Di Paolo, 2005; Thompson, 2007; Barandiaran et al., 2009; Louie, 2009, 2013, 2017a; Deacon, 2011; Montévil and Mossio, 2015; Moreno and Mossio, 2015; Mossio and Bich, 2017; DiFrisco and Mossio, 2020; Hofmeyr, 2021; Harrison et al., 2022; Mitchell, 2023; Mossio, 2024a).

This peculiar organization of living matter is both the source and the outcome of the capacity of a living cell or multicellular organism to *self-manufacture* (Hofmeyr, 2021). Life *is* what life *does*. A free-living cell, for example, must be able to produce all its required macromolecular components from external sources of matter and energy, must render these components functional through constant maintenance of a suitable and bounded internal milieu, must assemble functional components in a way that upholds its self-maintaining and self-reproducing abilities throughout its life cycle and, if we want it to evolve, must pass this integrated functional organization on to future generations via reproduction with some form of reliable but imperfect heredity (Saborido et al., 2011; Hofmeyr, 2021; Jaeger, 2024b; Pontarotti, 2024). This process of self-manufacture is encapsulated by the abstract concept of *autopoiesis* (Varela et al., 1974, 1991; Varela, 1979; Maturana and Varela, 1980; Weber and Varela, 2002; Thompson, 2007), which emphasizes the core ability of a living system to self-produce and maintain its own boundaries.

Biological organization emerged at the origin of life, and is therefore shared among all organisms, from bacteria to plants to fungi to animals to humans. In fact, some of us have argued previously that it is a fundamental prerequisite for biological evolution by natural selection (see Walsh, 2015; Jaeger, 2019, 2024b, for details). This kind of organization is unique to organisms. Nothing equivalent exists anywhere outside the realm of the living. While self-organizing physical processes, such as candle flames, convection currents (e.g., Bénard cells), turbulent water flows, or hurricanes, share some important properties with living systems, including the temporary reduction of entropy at the cost of the local environment, they are not able to self-manufacture in the sense described above (Kauffman, 2000; Deacon, 2011; Djedovic, 2020; Hofmeyr, 2021).

Two aspects of biological organization are particularly important here. First, note that functional *organization* is not the same as physical *structure*: the capability to self-manufacture does not coincide with any specific arrangement of material parts, nor does it correspond to any fixed pattern of interacting processes (cf. Rosen, 1991; Louie, 2009). Instead, the systemic pattern of interactions that constitutes biological organization is fundamentally fluid and dynamic: connections between components constantly change—in fact, *have* to change—for the capacity to self-manufacture to be preserved (see, for instance, Kauffman, 2000; Deacon, 2011; DiFrisco and Mossio, 2020; Hofmeyr, 2021; Jaeger, 2024b).

Second, at the heart of all contemporary accounts of biological organization lies the concept of *organizational closure* (see, Letelier et al., 2011; Moreno and Mossio, 2015; Cornish-Bowden and Cárdenas, 2020; Mossio, 2024b, for reviews). Organizational closure is a peculiar relational pattern of collective dependence between the functional components of a living system: each component process must be generated by, and must in turn generate, at least one other component process within the same organization (Montévil and Mossio, 2015; Moreno and Mossio, 2015; Mossio et al., 2016). This means that individual components could not operate—or even exist—without each other. Let us illustrate this central concept using two complementary approaches.

One useful way to think about biological organization is to separate the underlying *processes* (physico-chemical flows) from the higher-level *constraints* that impinge on them by reducing their dynamical degrees of freedom (Juarrero, 1999, 2023; Deacon, 2011; Pattee and Rączaszek-Leonardi, 2012; Montévil and Mossio, 2015; Mossio et al., 2016). Constraints arise through the interactions between the component processes that make up the living system. Like the underlying flows, they are dynamic, but change at different time scales. Constraints can thus be formally described as boundary conditions imposed on the underlying dynamics (Montévil and Mossio, 2015; Mossio et al., 2016). They decrease the degrees of freedom of the living system as a consequence of the restrictions that are placed upon it by the organized interactions of its constituent processes. An enzyme is a good example of a constraint: it alters the kinetics of a biochemical reaction without itself being altered in the process.

We can now conceptualize organizational closure as the *closure of constraints* (Montévil and Mossio, 2015): the organism-level pattern of constraints restricts and channels the dynamics of the underlying processes in such a way as to preserve the overall pattern of constraints. Evidently, organizational closure is causally circular: it is a form of *self-constraint* (Juarrero, 1999, 2023; Montévil and Mossio, 2015). In this way, the organization of the system becomes the cause of its own relative stability: this is what equips an organism with identity and individuality (Deacon, 2011; Montévil and Mossio, 2015; Moreno and Mossio, 2015; Mossio et al., 2016; DiFrisco and Mossio, 2020).

Organizational closure requires thermodynamic openness: it only occurs in physical systems that operate far from equilibrium. The basic reason for this is that the organism must constantly produce work by harvesting some entropy gradient in its environment to regenerate, repair, and replenish its set of constraints (Kauffman, 2000; Deacon, 2011). This leads to *organizational continuity* (DiFrisco and Mossio, 2020; Mossio and Pontarotti, 2020; Pontarotti, 2024). To revisit our previous example, think of all the enzymes in a living cell, enabling a set of interrelated biochemical flows that lead to macromolecular synthesis and the continued replenishment of the pool of enzymes. This requires physical work (driven by ATP-dephosphorylation, and other exergonic reactions). Note that enzyme concentrations need not be kept constant over time. They only have to remain within the less stringent boundaries that ensure the future preservation of overall metabolic flow. Metabolic flow can (and indeed must) change adaptively in response to environmental conditions and the internal requirements of the organism, but if it ceases to repair and replenish itself, the organism dies.

A more formal and abstract way to think about biological organization is Robert Rosen’s relational theory of *metabolism-repair (M,R)-systems* (Rosen, 1958a,b, 1959, 1972, 1991), and its recent refinement to *fabrication-assembly (F,A)-systems* (Hofmeyr, 2021). It treats biological organization in the rich explanatory context of Aristotle’s four “causes,” or *aitia* (Rosen, 1991; Louie, 2009, 2013, 2017a). Aitia go beyond our restricted sense of “causality” in the modern scientific sense. They correspond to different ways of answering “why” questions about some natural phenomenon (Falcon, 2023). As an example, take a marble sculpture depicting Aristotle: its *material cause* is the marble it is made of, its *formal cause* is what makes it a sculpture of Aristotle (and not of anyone else), its *efficient cause* is the sculptor wielding their tools to produce the sculpture, and its *final cause* is the sculptor’s intention to make a statue of Aristotle.

Using the (meta)mathematical tool of *category theory*, Rosen constructs a rigorous formal framework that distinguishes material causes (physico-chemical flows) from their *processors*, which are the efficient causes generating the particular dynamics that characterize a living system (Rosen, 1991, later extensively refined by Louie, 2009, 2013, 2017a). Thinking of enzymes again as possible examples of efficient causes, we can see that this distinction is similar in spirit, but not equivalent, to the separation of processes and constraints above. Constraints, as we shall see, not only incorporate aspects of efficient but also of formal cause.

**FIGURE 2:**
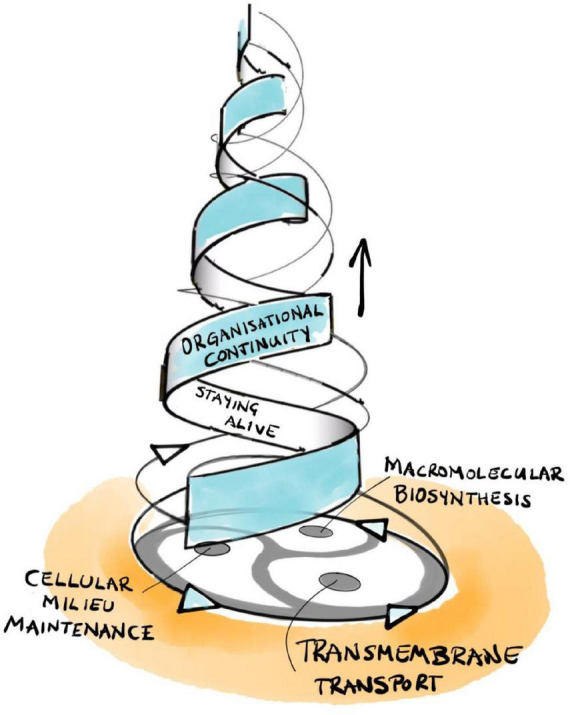
The triadic dialectic (trialectic) underlying cellular self-manufacture.

Rosen’s central insight is that his (M,R)-system models are open to material (and energy) flows but are *closed to efficient causation* (Rosen, 1991; Louie, 2009, 2013, 2017a).^11^ This is a form of organizational closure, meaning that each processor has as efficient cause another processor *within* the organization of the system. Formally, each processor must be part of a *hierarchical cycle of efficient causation*. Such cycles represent a type of self-referential circularity that Rosen calls *immanent causation*, which represents more than mere cybernetic feedback, the latter being restricted to material causes (i.e., hierarchically “flat”) and only generating circular *material* flows. Hierarchical cycles, in contrast, consist of nested cycles of interacting *processors* that preserve their own pattern of interrelations over multiple scales of space and time. As an example, recall the circular multi-level relationship between intermediate metabolism/macromolecular biosynthesis, the internal milieu, and the regulated permeability of the boundaries of a living cell that enable its self-manufacturing capability (Figure 2; Hofmeyr, 2017, 2021).

As a consequence of this hierarchical circularity, efficient cause coincides with final cause in living systems (Rosen, 1991). This is precisely what is meant by autopoiesis or self-manufacture: the primary and most fundamental goal of an organism is to keep on producing *itself*. Biological organization is intrinsically and unavoidably teleological in this specific and well-defined sense (Weber and Varela, 2002; Deacon, 2011; Mossio and Bich, 2017). We will discuss the wider consequences of introducing this particular kind of finality into the study of biological systems in section “8 Conclusion.” For now, let us simply assure the reader that it leads to none of the difficulties usually associated with teleological explanation (as argued in detail in Walsh, 2015).

Hofmeyr extends Rosen’s mathematical methodology in a number of crucial aspects. First, he integrates the missing formal cause into Rosen’s framework: its role is to determine the specific functional form of each efficient processor and/or material flow (Hofmeyr, 2018, 2021). It is in this precise sense that the notion of “constraint” includes aspects of both formal and efficient cause. With this tool in hand, we can now extend Rosen’s framework and map it explicitly onto the cellular processes involved in *self-manufacture*—intermediary metabolism/macromolecular biosynthesis, maintenance of the internal milieu, and transmembrane transport (Figure 2; Hofmeyr, 2017, 2021). The resulting model is called a fabrication-assembly (F,A)-system to reflect the fact that self-manufacture consists of two fundamental aspects: *self-fabrication* of required components, plus their *self-assembly* into a functional whole (Hofmeyr, 2007, 2017, 2021).

(F,A)-Systems highlight a number of features of biological organization that are not evident from Rosen’s original account. First of all, one of the major efficient causes of the model (the interior milieu) exists only at the level of the whole living system (or individual cell), and cannot be reduced or localized to any subset of component processes (Hofmeyr, 2007, 2017). If it was not already clear before: biological organization is an irreducible systems-level property. This is perhaps why it is so difficult to study with purely reductionist analytical approaches (but see Jaeger, 2024c).

Second, (F,A)-systems are closed to efficient cause but *open to formal causation.* This means that the specific form of their processors and flows constantly changes while still maintaining organizational closure (Hofmeyr, 2021). This enables physiological and evolutionary adaptation by introducing *heritable variability* to Rosen’s formalism (see also Barbieri, 2015). It links the fundamental biological principles of organization and variability in a way that is not possible with the less refined distinction between processes and constraints (cf. Montévil et al., 2016; Soto et al., 2016a,b; Jaeger, 2024b). We will revisit this topic in section “6 Affordance, goal, and action,” when we start focusing on ecology and evolution.

Finally, due to overall closure all efficient causes in the (F,A)-model are part of *hierarchical cycles* with the mathematical property of being self-referential in the sense of being *collectively impredicative*, which means that the processors involved mutually define and generate each other, and thus cannot exist in isolation (Hofmeyr, 2021). This leads to an apparent paradox: without these processors all being present at the same time, already interacting with each other, none of them can ever become active in the first place. To compare and contrast it with Turing’s (1937) *halting problem*, Hofmeyr calls this kind of deadlock the *starting problem* of biological systems. It illustrates the basic difference between mere recursion (processes feeding back on each other) and co-construction (processes building each other through continuous constraint-building).^12^

With this conceptual toolkit in hand, we can now revisit and refine the distinction between living and non-living systems, in order to better understand why the self-manufacturing organization of living matter cannot be fully formalized or captured by algorithmic computation. Rosen frames this problem in terms of complexity (Rosen, 1991; see also Louie, 2009; Louie and Poli, 2017; Hofmeyr, 2021). He (re)defines a *complex system* through the presence of at least one hierarchical cycle among its functional components, while the category of simple systems comprises all those that are not complex (Rosen, 1991; Louie, 2009). Rosennean complex systems include living cells and organisms, plus systems that contain them (such as symbioses, ecologies, societies, and economies). This contrasts with more familiar definitions of “complexity,” which rely on the number, nonlinearity, and heterogeneity of (interactions among) system components, on the presence of regulatory feedback and emergent properties, and/or on the algorithmic incompressibility of simulated system dynamics. Such definitions result in graded rather than categorical differences in the complexity of living vs. non-living systems (see, for example, Mitchell, 2009; Ladyman and Wiesner, 2020).

Based on his categorical distinction between simple and complex systems, Rosen derives his most famous conjecture (Rosen, 1991; Louie, 2009, 2013, 2017a): he shows, in a mathematically rigorous manner, that only simple systems can be captured completely by analytical (algorithmic) models, while any characterization of complex systems in terms of computation must necessarily remain incomplete. This closely relates to our claims concerning the formalization of relevance realization from section “3 Relevance realization.” Rosen’s, like ours, is an incompleteness argument analogous to Gödel’s proof in mathematics. It says that it may well be possible to *approximate* aspects of biological organization through algorithmic simulation, but it will never capture the full range of dynamic behaviors or the evolutionary potential of a living system *completely*. If true, this implies that the strong Church-Turing conjecture—that all physical processes in nature must be computable (Deutsch, 1985, 1997; Lloyd, 2006; see section “2 Agential emergentism”)—is false, since biological organization provides a clear counterexample of a physical process that cannot be captured fully by computation.

Here, we extend and recontextualize Rosen’s conjecture to arrive at an even stronger claim: *it no longer makes sense to ask if organisms are computable if they are not completely formalizable* in the first place. While discussions about computability focus on our limited ability to *predict* organismic behavior and evolution, our argument about formalization reveals even deeper limitations concerning our ability to *explain* living systems.

To recognize these limitations for what they are, we need to return to the matter of intrinsic goals. Once a living system is able to maintain organizational continuity through self-constraint or immanent causation, it starts exhibiting a certain degree of *self-determination* (Mossio and Bich, 2017). In other words, it becomes *autonomous* (Moreno and Mossio, 2015) because, ultimately, the process of maintaining closure must be internally driven. Even though the environment is a necessary condition for existence (not just as a source of food and energy, as we shall see in section “6 Affordance, goal, and action”), an organism does not behave in a purely reactive manner with regard to external inputs. Instead, future states of the system are *dynamically presupposed* by its own inherent organization at earlier points in time (Bickhard, 2000). This is exactly what we mean when we say an organism is its own final cause. If organizational continuity ceases, the organism dies and is thus no longer a living system.^13^ It still engages in exchange with its environment (e.g., by getting colonized by saprophages), but the inner source of its aliveness is gone. According to Rosen, it has made the transition from complex to simple. Complexity, therefore, originates from within. And it remains opaque to any external observer, forever beyond full formalization.

Taken together, all of the above defines in a minimal account of what it means for an organism to act for its own reasons, on its own behalf (Kauffman, 2000; Mitchell, 2023): *basic natural agency* is characterized by the ability to define and attain the primary and principal goal of all living beings—to keep themselves alive. This is achieved through the process of autopoiesis or self-manufacture, implemented by a self-referential, hierarchical, and impredicative causal regime that realizes organizational closure. This simple model, which is completely compatible with the known laws of physics, provides a naturalistic proof of principle that organisms *can* (and indeed *do*) pursue at the very least one fundamental goal: to continue their own existence. It accounts for the organism’s fundamental *constitutive autonomy* (Moreno and Mossio, 2015). But this is not enough. What we need to look at next is another important dimension of an organism’s behavior: its agent-arena relations, which are guided by a kind of *interactive autonomy* (Di Paolo, 2005; Moreno and Mossio, 2015; Vervaeke et al., 2017). A full-blown account of natural agency requires both organizational and ecological dimensions. The latter will be the topic of the next two sections.

## 5 Basic biological anticipation

The interactive dimension of natural agency is also called *adaptive agency*, because it is concerned with how an organism, once it has achieved basic self-manufacture, can adaptively regulate its state in response to its environment (Di Paolo, 2005; Moreno and Mossio, 2015; Di Paolo et al., 2017). As an example, consider the paramecium again: its cilia beat as a consequence of its metabolism and the maintenance of its internal milieu, but their effect lies outside the cell, causing the organism to move toward food sources, or food to be brought into proximity through the turbulent flow induced in its viscous watery surroundings. Thus, constraints subject to closure can (and indeed must) exert effects beyond the boundaries of the organism.^14^ *Agency is* not only an organizational, but also *an ecological phenomenon* (Walsh, 2015). It is as much about the relations of the agent to its arena, as it is about internal self-manufacture.

Once it has set itself a goal, the organism needs to be able to pursue it. Such pursuit presupposes two things: first, the organism must be *motivated* to attain its goal. Motivation ultimately springs from an organism’s fragility and mortality (Jonas, 1966; Weber and Varela, 2002; Thompson, 2007; Deacon, 2011; Moreno and Mossio, 2015; see also section “2 Agential emergentism”). If a system cannot perish (or at least suffer strain or damage), it has no reason to act. This is why the execution of an algorithm must be triggered by an external agent. It pursues nothing on its own.^15^

Second, and more importantly, the organism must be able to identify appropriate combinations or sequences of actions, suitable strategies, that increase the likeliness of attaining its goals. This is what it means to make the right “choice”: to select an appropriate action or strategy from one’s repertoire that satisfices in a given situation. The chosen path may not be optimal—nothing ever is. But making adequate choices still requires the ability to assess, in some reliable way, the potential consequences of one’s actions. More precisely, it means that even the simplest organism must have the capacity to project the present state of the world into the future or, perhaps more appropriately, to pull the future back into the present (Louie, 2009; Rosen, 2012; Kiverstein and Sims, 2021; Sims, 2021). Or simply put: any purposive system is able to perform an activity (rather than some other activity) because of its (likely) consequences. Recall that this cannot rely on intention or awareness, if we are to develop an account of relevance realization suitable for *all* living beings. Therefore, our next task is to show that even the most primitive organisms are *anticipatory systems* (Louie, 2009, 2012, 2017b; Rosen, 2012).

An anticipatory system possesses *predictive internal models* of itself and its immediate environment (the accessible part of the large world it lives in; Rosen, 2012). These models need not be explicit representations. They simply stand for an organism’s “expectations” in some generic way. Often, they manifest as evolved automatisms or habituated instinctive behaviors: selectors of actions inherited from ancestors that attained their goals to survive (and later reproduce) in comparable circumstances in the past.^16^ Only exceptionally, in a small set of highly complex animals (including humans), can we speak of representations or even premeditated mental simulations of future events (Deacon, 2011). Most internal predictive models are much simpler, yet still anticipatory. Let us examine what the minimal requirements of such biological anticipation are.

To start with a simple example: a bacterium can modify the frequency of change in its flagellar motion to swim up a nutrient gradient or, conversely, to avoid the presence of a toxin. This evolved automatism anticipates the outcome of the bacterium’s actions in two ways: first, it enables the organism to discern between nutritious (good) and noxious (bad) substances in its surroundings. Second, it directs the bacterium’s movements toward an expected beneficial outcome, or away from a suspected detrimental one. This process can and does go wrong: outside the laboratory there is not always an abundance of food at the top of the sugar gradient, and new dangers constantly await.

It is important to repeat that there is nothing the bacterium explicitly intends to do, nor is it in any way aware of what it is doing, or how it selects an appropriate action. Its responses are evolved habits in the sense that there are few alternative paths of action, there is very little flexibility in behavior, and there is certainly no self-reflection. And yet, bacteria *have* evolved the capacity to distinguish what is good and what is bad for their continued existence, purely based on endless runs of trial-and-error in countless generations of ancestors. This is basic relevance realization grounded in adaptive evolution. And it qualifies as basic anticipatory behavior: the expected outcome of an action influences the bacterium’s present selection of actions and strategy. The fundamental requirement for a predictive model is fulfilled: there is a subsystem in the bacterium’s physiology that induces changes in its present state based on expectations about what the future may bring. This is what we mean when we say that anticipatory systems can pull the future into the present.

More generally, we can consider a living system organized in the way described in section “4 Biological organization and natural agency,” which contains predictive models as subsystems with effectors that feedback on its self-manufacturing organization. These effectors influence system dynamics in two distinct ways (Louie, 2009, 2012, 2017b; Rosen, 2012). Either they modify the selection of actions (as in the bacterial example above), or they modulate the sensory inputs of the system (see also Kiverstein and Sims, 2021). Our expectations constrain and color what we perceive. It is in this sense that anticipation is most essential for relevance realization.

Let us consider human predictive processing once more (see section “3 Relevance realization”): on the one hand, people cannot help but project their expectations onto their environment. It is hard to let go of preconceived notions: we only modify our expectations when we encounter errors and discrepancies of a magnitude we can no longer ignore. On the other hand, we also use opponent processing to great effect when we play predictive scenarios against each other while monitoring the streams of discrepancies they generate to prioritize between them. We will revisit such adaptive evolution of internal models in section “7 To live is to know.” For now, it suffices to say that human beings are anticipatory systems par excellence, and our behavior cannot be understood unless we take seriously our abilities to plan ahead and strategize.

**FIGURE 3:**
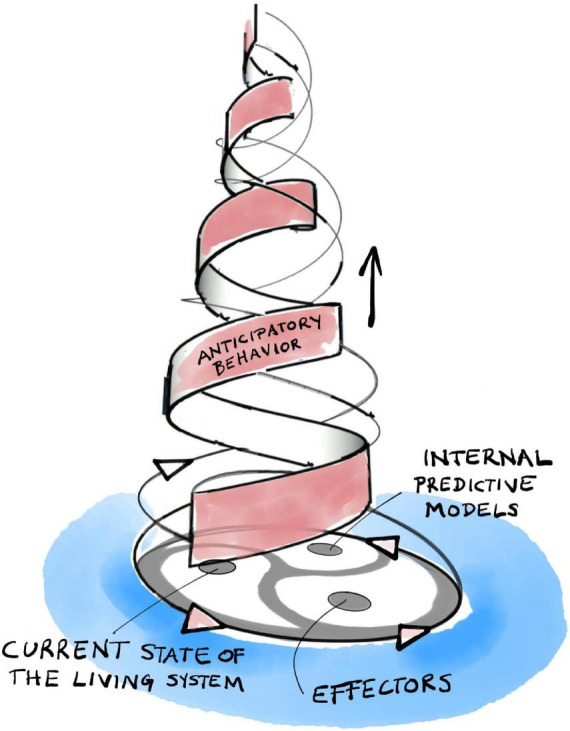
The triadic dialectic (trialectic) underlying anticipatory systems.

The internal organization of anticipatory systems is intimately related to the basic organization of living systems (Louie, 2012; Rosen, 2012). There are familiar self-referential patterns (Figure 3). Without going into too much detail, let us note that organisms generate their internal models of the world from within their own organization. These models, in turn, direct the organism’s behavior, its choice of actions and strategies, through their effectors. Actions have consequences and, thus, our internal models become modified through the comparison of their predictions with actual outcomes. This leads to a dialectic adaptive dynamic guiding our active explorations of a large world, not unlike that which governs the interactions of self-manufacturing processes described in section “3 Relevance realization” (compare Figure 2 with Figure 3).

As is the case with Rosennean complex systems, there are anticipatory systems that are *not* organisms. Economies, as a case in point, are heavily driven by internal (individual- and societal-level) models of their own workings (e.g., Tuckett and Taffler, 2008; Schotanus et al., 2020). Once somebody finds out how to predict the stock market, for instance, its dynamics will immediately and radically change in response. We can thus say that all organisms are anticipatory systems, but not all anticipatory systems are organisms; similarly, all anticipatory systems are complex systems, but not all complex systems are anticipatory (Louie, 2009; Rosen, 2012).

To summarize: all organisms, from bacteria to humans, are anticipatory agents. They are able to set their own goals and pursue them based on their internal predictive models. *Organisms, essentially, are systems that solve the problem of relevance.* In contrast, algorithms and machines are purely reactive: even if they seemingly *do* anticipate and are able to simulate future sequences of operations in their small-world context, it is always in response to a task or target that is ultimately predefined and externally imposed.

It is worth interjecting at this point that nothing in our account violates any laws of logic or physics. In particular, we do not allow future physical states of the world to affect the present. The states of internal predictive models are as much physically embedded in the present as the organism of which they are a part. Also, the model can (and often does) go wrong. Consequences of actions are bound to diverge from predictions in some, often surprising and unexpected, ways. A good model is one where they do not diverge catastrophically. A bad model can be improved by experience through the monitoring and correction of errors. This yields the adaptive dialectic dynamic depicted in Figure 3. Remember that every living system can do this. With this capacity in hand, we can now move to the bigger picture of how organisms evolve an ever richer repertoire of goals and actions through solving the problem of relevance (cf. Roli et al., 2022).

## 6 Affordance, goal, and action

We now come to the core of our *evolutionary account of relevance realization*, which is based on an organism-centered agential perspective on evolution called *situated Darwinism* (Walsh D. M., 2012, 2015). It is *an ecological theory of agency and its role in evolution*, which centers around the engagement of organisms with their experienced environment. Situated Darwinism centers around the following three basic ingredients: (1) a collection of *intrinsic goals* for the organism to pursue (see section “4 Biological organization and natural agency”), (2) a set of available actions (the *repertoire* of the agent, shaped with respect to its experience and expectations; section “5 Basic biological anticipation”), and (3) *affordances* in the experienced environment. In what follows, we show that the dialectic co-emergent dynamics between these three components provide an *evolutionary explanation of relevance realization.*

*Affordances* are what the environment offers an agent (Gibson, 1966, 1979; Chemero, 2003; recently reviewed in Heras-Escribano, 2019). They typically manifest as opportunities or impediments, defined with respect to an organismic agent pursuing some goal (Walsh, 2015). An open door, for example, affords us to pass through it, but when the door is shut it prevents us from entering. Similarly, gradients of nutrients and toxins provide positive and negative affordances to a bacterium seeking to persist and reproduce. In this context, perception becomes the active identification of affordances. Actions are initiated in direct response to them. Affordances are a quintessentially relational, ecological, and thus transjective phenomenon: they do not reside objectively in the physical environment, but neither are they subjective. Instead, they arise through the exploratory interaction of an agent with particular attributes and aspects of its surroundings (Chemero, 2003; Stoffregen, 2003; Di Paolo, 2005; Walsh, 2015). They are what constitutes the agent’s *arena*, the task-relevant part of its experienced environment (see section “3 Relevance realization”).

**FIGURE 4:**
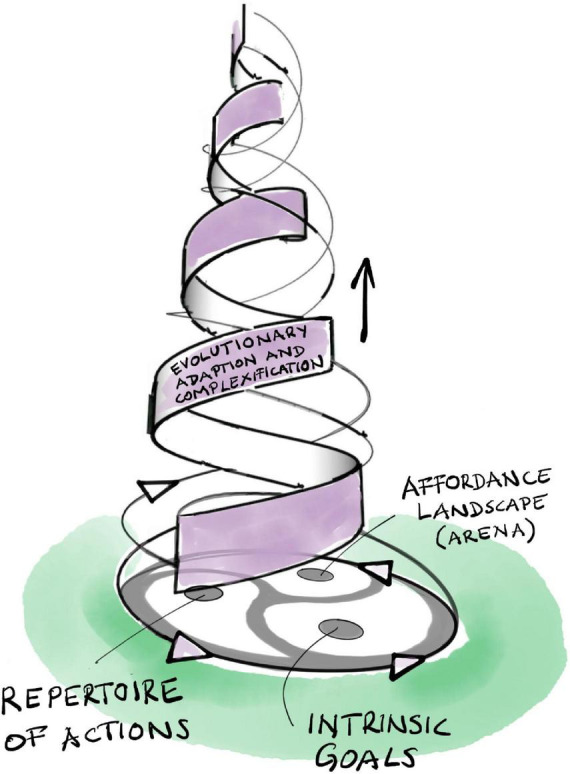
The triadic dialectic (trialectic) underlying evolutionary adaptation and complexification.

Affordances ground the agent-arena relationship in the world: organisms experience their physical environment as an *affordance landscape* (Walsh, 2014, 2015; Felin and Kauffman, 2019). This concept highlights the complementarity of agent and arena. The latter does not simply preexist, independent of the agent. Through their mutual interrelations, an organism’s goals, actions, and affordances continuously co-constitute each other through the kind of emergent dialectic dynamic we have already encountered in the last two sections (Figure 4). The arena (as an affordance landscape) constantly co-emerges and co-evolves with the evolving set of goals and action repertoire of the agent as it explores and comes to know the large world it is living in.

This dialectic proceeds in the following way: an organismic agent identifies affordances in its surroundings through active sensing or perception (which may be based on predictive processing as described in section “3 Relevance realization”), generating its arena by delimiting the task-relevant region of its larger experienced environment. This highlights the co-dependency of agent and arena: an organism’s affordance landscape exists only in relation to the set of intrinsic goals that the organism may select to pursue^17^ (see section “5 Basic biological anticipation”). This landscape represents a world of meaning, laden with value, where affordances are more or less “good” or “bad” with respect to attaining the agent’s goals. We cannot repeat often enough that this does not require any explicit intention, awareness, or cognitive or mental processing. The classification of affordances as beneficial or detrimental, and their effect on the process of selecting a goal, may be habituated by adaptive evolution through the inherited memory of the successes and failures of previous generations.

To complicate the situation, different (even conflicting or contradictory) goals can coexist at the same time, even in agents with very simple behavioral repertoires and predictive internal models. A bacterium, for instance, may encounter a nutrient gradient that coincides with the presence of a toxin, contradictory affordances which create conflicting signals of attraction and avoidance. Moreover, the same goal can be assigned different weights (priorities) in different situations, and goals may build on each other in other non-trivial ways that tend to be radically dependent on context. All of this means that the goals of an agent come with a complicated and rich structure of interdependencies, intimately commingled with the affordances in its arena (Haugeland, 1998; Walsh D. M., 2012; Walsh, 2014, 2015).

When an agent selects a particular goal to pursue, it collapses its set of goals down to one specific element. Similar considerations apply to the choice of an appropriate action, or a small set of actions that constitute a strategy, from the organism’s current repertoire to pursue the selected goal. This repertoire also comes with a complicated and rich internal structure: some actions are riskier, or more strenuous, or more difficult to carry out than others; some are quicker, more obvious, or more directly related to the attainment of the goal. Some actions only make sense if employed in a certain combination or temporal order. The potential usefulness of an action must be assessed through internal predictive models that take all these complications into account (see section “5 Basic biological anticipation”). Based on this, the agent collapses its repertoire to select a particular action, or combination or sequence of actions, which it will commit to carry out.

All of this leads to the agent leveraging specific positive affordances in its arena, while trying to avoid or ameliorate negative ones, through its actions thereby also collapsing the set of available affordances (the arena) to some subset of itself. Taken together, all of the above results in a constant, coordinated, and co-dependent collapse and reconstruction of all three sets—affordances, goals, and actions—ultimately committing the agent to a particular pursuit at any one time through a specific sequence of actions. This is how agential behavior is generated.

Of course, this is not the end of the process. Committing to a specific action or strategy will immediately change the affordance landscape and will likely also affect the set of goals of the agent. Acting in one’s arena inevitably generates new opportunities and impediments, while modifying, suppressing, or removing others. The altered affordance landscape is then again perceived and assessed by the agent (through its internal predictive models), affecting the weights and relations of its goals, while generating new ones (and obsoleting others). All the while, the agent may learn to carry out new actions and to adjust old ones. This iterative evaluation and amelioration of behavioral patterns (Figure 4) leads to *an adaptive dynamic* that induces a closer fit between the agent and its arena, a tighter and often more intricate agent-arena relationship, and thus a firmer and broader grip on the world. In short, the agent may learn to behave more appropriately in its particular situation. It is enacting its own adaptation (Di Paolo, 2005).

This yields *an organism-centered model of Darwinian evolution*, which is very different from most other current approaches to evolutionary biology. It is thoroughly Darwinian, because the population-level selection of heritable variants remains central for stabilizing adaptive behavioral patterns across generations (see Walsh D. M., 2012; Walsh, 2015). This is especially evident in simple organisms (like bacteria) with small and rather inflexible sets of affordances, goals, and actions that show a high degree of genetic influence and low flexibility in their behaviors. Yet, unlike reductionist and strictly adaptationist accounts of Darwinian evolution, it also provides an explanation for how such simple behaviors can evolve into more complex and multi-layered interactions between an agent and its arena over time.^18^

Situated Darwinism implies that agents and arenas constantly co-evolve and co-construct each other (Walsh, 2014, 2015; Rolla and Figueiredo, 2023). This constant mutual engagement recapitulates Darwin’s original framing of evolution through the organism’s *struggle for existence*. In particular, it reunites the selection of appropriate behaviors by an agent during its lifetime (physiological and behavioral adaptation) with the organism’s ability to preserve and adjust its organization within and across generations (evolutionary adaptation; Saborido et al., 2011; DiFrisco and Mossio, 2020; Mossio and Pontarotti, 2020; Pontarotti, 2024). Several of us have argued in detail elsewhere that such an integration of adaptive processes within and across generations is a fundamental prerequisite for evolution by natural selection (Walsh, 2015; Jaeger, 2019, 2024b).

**FIGURE 5:**
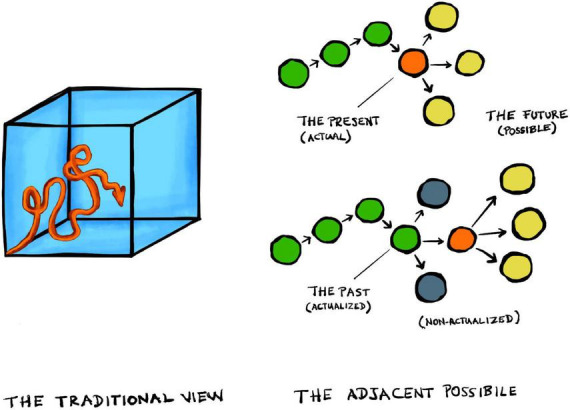
Two differing views on evolutionary possibilities.

The account we present here provides a powerful framework for thinking about evolutionary innovation and the generally open-ended nature of evolutionary dynamics [as already argued in Roli et al. (2022) and Jaeger (2024b)]. Its underlying co-emergent and co-constructive dialectic grounds the notion of the *adjacent possible* in autopoiesis, anticipation, and our integrated concept of adaptation (Kauffman, 2000; see also Di Paolo, 2005). Think of an affordance landscape (at a specific time) as a map of possible actions/outcomes for an organism. Some of these actions/outcomes are not static or prespecified: as organisms respond to affordances, they alter the structure or topography of the affordance landscape. What was once improbable, may become highly attainable (and vice versa). This provides an alternative to the widespread idea that evolution happens in a predefined space of possibilities which, though astronomical in size, can be prestated (i.e., precisely circumscribed) *before* any evolution has actually taken place (Figure 5, left; see also Felin and Kauffman, 2019; Roli et al., 2022). The adjacent possible, in contrast, shows us that the box representing this space simply does not exist. It sees evolutionary possibilities as co-emerging with evolution, the adjacent possible being the space that contains everything that could actually happen next, given the current state of the world (Figure 5, right). This space is in constant flux, generated by the evolutionary process as it goes along. This leads to a radically open-ended view of evolution, in which possible future affordances, goals, and actions cannot possibly be prestated as well-defined sets ahead of them actually being jointly actualized (Roli et al., 2022). Kauffman (2000) calls this *radical emergence*.

It should be fairly evident from what we said so far that *situated Darwinism provides nothing other than an evolutionary reformulation and extension of relevance realization* as originally defined in the context of human cognition. It explicates the fact that all organisms, from bacteria to humans, are able to solve the problem of relevance by identifying and leveraging affordances in their arena according to their abilities and in pursuit of their intrinsic goals. Organismic agents must delimit their arena in a large world before they can engage in any kind of formal problem-solving (cf. section “3 Relevance realization”). This they achieve through the situated adaptive Darwinian process outlined above, which is able to generate more adequate and tighter local agent-arena relationships that are conducive to the agent’s goal of surviving and thriving in its particular environment. Relevance realization *is* this meliorative evolutionary dynamic. It is, at its very core, an adaptive and constructive process.

## 7 To live is to know

A multifaceted and multilayered picture of relevance realization in living organisms is emerging. What we have so far are three different dialectic processes, at three different levels of organization:

1. the process of *autopoiesis* (*self-manufacture*)—internal to the organism, established through collective co-constitution of macromolecular biosynthesis, maintenance of internal milieu, and regulated selective cross-boundary transport—which enables the agent to autonomously set its own intrinsic goals through self-determination (self-constraint, see section “4 Biological organization and natural agency”; Figure 2);
2. the process of *anticipation*—internal to the organism, but projective (about the environment), established through collective co-constitution of internal predictive models (“expectations”), the current state of the organism, and effectors modulating this state and the sensory inputs that feed it based on model predictions—which enables the agent to pursue intrinsic goals through selection of suitable actions and behavioral strategies (see section “5 Basic biological anticipation”; Figure 3); and
3. the process of integrated *adaptation*—transjective (grounded in the relation between agent and arena), established through collective co-constitution of the intrinsic goals, repertoires of action, and affordance landscapes of an organism-environment system—which amounts to *relevance realization in its broadest evolutionary sense*, a continuous tightening of the agent-arena relationship and hence the organism’s “grip on reality” (see section “6 Affordance, goal, and action”; Figure 4).

**FIGURE 6:**
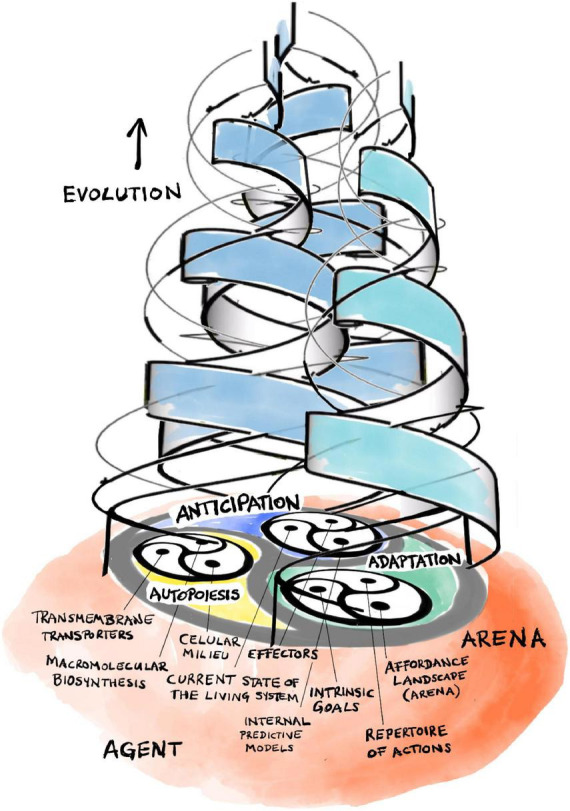
The hierarchical tangle of dialectic triads (trialectics) underlying open-ended evolution.

What remains to be done is to join these three levels of dialectic dynamics into a unified hierarchical model of open-ended organismic evolution, which explains how the different processes fit together to produce phenomena and behaviors of increasing adaptivity and complexity, including cognition and ultimately also consciousness (Figure 6; see also Juarrero, 1999, 2023; Deacon, 2011; Walsh, 2015; Roli et al., 2022; Mitchell, 2023; Jaeger, 2024a).

The first step toward such a multi-level integration is the realization that all three processes share *the same kind of underlying dialectic dynamics*, in which a triad of interrelated aspects of the process collectively generate and uphold each other to produce a unified, constructive, adaptive, and open-ended higher-level dynamic (Figures 2–4, summarized in Figure 6). This kind of dynamic is equivalent, at a certain degree of abstraction, to the triadic interaction between sign, object, and interpretant in the theory of *semiosis* originally proposed by pragmatic philosopher C. S. Peirce in his “Logic of Signs” (reproduced as chapter 3 of Favareau, 2010). We will call this kind of Peircean triadic dialectics “trialectics” for short. Let us emphasize once again that this trialectic dynamic can only be emulated or mimicked (but not captured completely) by recursive algorithmic simulation, due to its collectively impredicative and physically embedded organization (starting and halting problems, see section “4 Biological organization and natural agency”), its anticipatory rather than reactive nature (see section “5 Basic biological anticipation”), and its fundamental lack of prestatability and radical emergent open-endedness due to its emergent, co-constructive nature (expressed by the notion of the adjacent possible, see section “6 Affordance, goal, and action”).

What we need to do next is to show that this seemingly paradoxical trialectic can be scientifically justified—that it does not contradict any well-established empirical knowledge about the physical or the living world. What we want, after all, is a *naturalistic* account of relevance realization. It requires no mysterious new forces or fundamental laws of physics. All that is needed is a simple shift of focus, away from the predictive laws governing state change in the underlying physico-chemical processes to the unpredictable historical succession of dynamic constraints (further refined into a combination of closed efficient and open formal causes, see section “4 Biological organization and natural agency”), which are imposed on these processes in a living system, continuously restricting and channeling their degrees of freedom as well as their rate and direction of change (Polanyi, 1968; Anderson, 1972; Juarrero, 1999, 2023; Pattee, 2001; Deacon, 2011; Pattee and Rączaszek-Leonardi, 2012; Hofmeyr, 2018, 2021; Mitchell, 2023).

The *continuous collective co-constitution of constraints* is what unites the three levels of trialectic dynamics summarized above. It occurs under the fundamental condition (or we could say: within the meta-constraint) of having to continuously maintain closure of constraints (see section “4 Biological organization and natural agency”). It is what really matters when we try to understand life and its evolution. It is what generates and maintains the closed organization of a cell or organism, its ability to anticipate, and the adaptive and constructive physiological, behavioral, and population-level processes that not only tighten the agent-arena relationship, but also account for the evolutionary trend toward increased complexification. It is this process of increasingly intricate mutual constraint construction that differentiates living from non-living systems, allows them to evolve by natural selection (Jaeger, 2024b), and lends them coherence across multiple levels of organization, successively emerging through major transitions in evolution (Maynard Smith and Szathmáry, 1995; Szathmáry, 2015).

On this view, the functional capacities and evolutionary potential of organismic agents are fundamentally unpredictable and radically open-ended (Roli et al., 2022; Jaeger, 2024b). Biological organization is only enabled but not predetermined or driven by the underlying dynamical laws of physics whose possible outcomes it restricts. An organism’s individual life history and its evolution are full of consequential accidents (Polanyi, 1968; Pattee, 2001; Pattee and Rączaszek-Leonardi, 2012; Ruiz-Mirazo and Moreno, 2012). Due to an organism’s openness to formal cause (see section “4 Biological organization and natural agency”), evolution constantly explores new structural variants within the general confines of closure to efficient causation (Hofmeyr, 2018, 2021). This enables evolving agents to interact with their arena in truly unprecedented ways (Walsh, 2015). This does not have to happen frequently, it just has to be possible in principle for the future propensities of evolution to become radically uncertain: we cannot prestate them as an explicit probability distribution and, in fact, we cannot formalize them as a well-defined space of possibilities at the present time (see section “3 Relevance realization”; Kauffman, 2000; Roli et al., 2022).

To understand better what this means, it is useful to look at the process of constraint construction from two complementary perspectives. So far, we have relied on a *constitutive frame*: it begins with the positive observation that each of the three aspects of a trialectic constraint-generation process is absolutely necessary to bring about and uphold the other two. Even though each subprocess is distinguishable and describable in its own terms, they can never be physically separated or occur in isolation from each other. They must emerge from each other. They would not even exist without one another’s mutual support. They always *have* to actualize together. It is in this sense that they are collectively impredicative, the presence of one necessarily entailing the presence of the other two (see section “4 Biological organization and natural agency”), and that they are also concurrent, in exchange with each other all of the time (see section “6 Affordance, goal, and action”). In summary: the constitutive perspective focuses on the interrelations between physico-chemical processes that are simultaneously *present* to explain overall dynamics.

In a complementary way, we can focus on what is *not* there but nevertheless exerts causal influence on organismic dynamics (Deacon, 2011; Pattee and Rączaszek-Leonardi, 2012). We can call this approach the *constraint frame*. It seems perplexing at first sight. Think of it as the photographic negative of the constitutive angle. As we shall see, it turns out to be extremely useful for our purposes. Taking the constraint perspective begins with the observation that each subprocess in a trialectic enables reciprocal support by establishing a coherence among the different aspects of the overall process. More specifically, the emergence of coherent overall dynamics requires *reducing* the degrees of freedom of each individual subprocess, to ensure that all three fall into a regime that allows them to interact in a mutually supportive, constructive, and generative manner. The whole living organism, in this sense, is *less* than the sum of its parts (Deacon, 2011).

**FIGURE 7:**
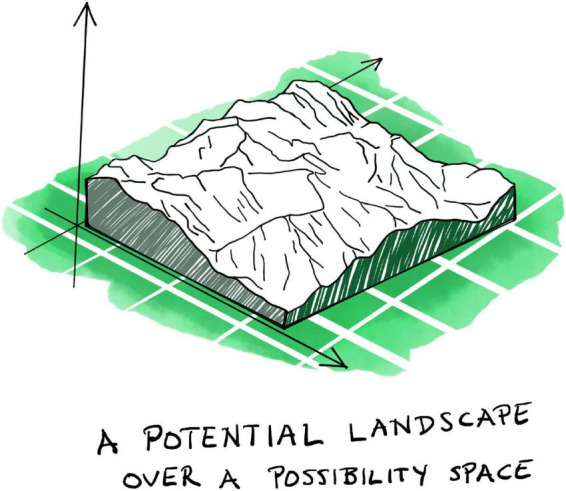
Shape vs. accessibility—constitutive vs. constraint perspectives.

It may be useful to visualize the complementary nature of the two frames or perspectives in the following way. A physico-chemical process, given its current state and structure, always has a distribution of future probabilities associated with it—determined by a space of possibilities that in dynamical systems theory is called the *configuration space* of the system (see, for example, Jaeger et al., 2012; Jaeger and Monk, 2014). This abstract space can be represented as a (usually very high-dimensional) distribution of probabilities, and can be pictured as a landscape in which the likelihood of possible system configurations is shown as altitude, with lower-lying configurations (valleys or troughs in the landscape) being the more probable ones to be actualized (Figure 7). The constitutive perspective aims to explain how this landscape is *shaped* in the first place through collectively co-constitutive processes. In contrast, the constraint perspective aims to understand which parts of the topography are actually accessible from the current state and permissive at the same time—in the sense of being conducive to the continued maintenance of closure. Both perspectives are needed to understand the peculiar dynamics and evolution of living matter.

As a second step toward grounding and integration of the three levels of trialectic dynamics shown in Figure 6, we need to explore the broader physical context in which constraint generation can actually occur. To stay alive and continue to evolve and explore the world is not something that happens spontaneously to an organism. It requires physical work—specifically, the kind of work that can be used to maintain and generate the constraints necessary to keep the organism going (Kauffman, 2000; Deacon, 2011). Kauffman (2000) calls this the *work-constraint cycle*: free energy is channeled into work that (instead of just dissipating the energy) builds and maintains a set of constraints, which in turn channel and direct more work required to build and maintain further constraints, and so on (see also Roli et al., 2022).

At first sight, it may seem strange that work should be required to do *less*, to *restrict* the range of possible dynamical behaviors of a component process.^19^ To understand what is going on in physical terms, it helps to conceptualize constraints as a measure of *orderliness* (Deacon, 2011): the more constraints are present in a system, and the more intricate and specific their effects and interactions, the lower the system’s entropy will be. Maximum entropy is reached when everything is possible; maximal degrees of freedom imply an absence of constraints. That is why generating constraints (i.e., restricting dynamical behavior) takes physical work: it means lowering the entropy of the system. This can only happen in systems able to tap into an entropy gradient as an external source of free energy. This source is what gives the organism the capacity to do physical work (Kauffman, 2000; Deacon, 2011). Obviously, the only systems for which this is possible are those that are thermodynamically open, operating far from equilibrium.

Last but not least, we need to revisit an important distinction: biological organization is not the same as mere *self-organization* in non-living dissipative systems such as eddies, convection cells, hurricanes, or candle flames, which burn through their source of free energy at the maximum possible rate (Deacon, 2011). The increased internal orderliness of such systems is bought at the cost of maximal disruption in their local environment. Think of a tornado wreaking havoc in its path. Life is different. The continuous mutual co-constitution of constraints extends the state or orderliness for as long as possible by maximizing both the rate of energy dissipation *and* the path length toward the inevitable depletion of the local entropy gradient (Deacon, 2011; Djedovic, 2020). This can occur through the exploration of new energy sources (e.g., foraging or growth toward light), or through storage and reuse (e.g., fat or polysaccharide deposits).

All of this leads to the coherent—but still historically contingent and unpredictable—constructive trialectic dynamic across multiple levels of organization that is shown in Figure 6. In fact, there is nothing in this scheme that limits the emergence of new kinds of organization. We can now put a concrete meaning to William Wimsatt’s definition of levels as “local maxima of regularity and predictability in the phase space of alternate modes of organization of matter” (Wimsatt, 2007, p. 209). A new level of organization arises through the emergence of a coherent and self-sustaining dynamic of the general kind described above. This process is driven by physical work (the harvesting of some entropy gradient in a thermodynamically open, far-from-equilibrium system) performed at a lower level of organization (Deacon, 2011). Obviously, the three kinds of organization described above are not the only conceivable ones. The emergence of higher levels is not only possible but extremely likely to occur in this account of radically open-ended evolution.

What could such higher levels of dynamic organization represent? Generally, their emergence is marked by major evolutionary transitions, which bring additional levels of organization into play (Maynard Smith and Szathmáry, 1995; Deacon, 2011; Szathmáry, 2015). As an example, consider the sympoiesis at the origin of the eukaryotic lineage: the constructive emergence of an integrated organization from the merger of two formerly independent organisms, which enabled a much richer behavioral repertoire (West et al., 2015). The protensive explorations of an amoeba with its pseudopods represents a much more sophisticated kind of goal-orientedness than the random tumblings of the bacterium.

Of particular interest here are two scenarios that build on each other. The first occurs when the interrelations between goals, actions, and affordances surpass a certain threshold of intricacy, such that they require a new kind of predictive internal model. This yields a definition of a *cognitive system* as an agent which can actively take its world to be a certain way, regardless of whether the world really is that way or not (Corcoran et al., 2020; Djedovic, 2020). In other words, a cognitive system is an agent that is complex enough to be mistaken or deluded about the world. While a bacterium may fail to achieve its aim of finding higher concentrations of nutrients when swimming up a concentration gradient (the distribution could be discontinuous, or other unexpected dangers may lurk at the top of the gradient), it is too simple to have a wrong model of the world. The failure modes of bacteria and cognitive systems diverge in this instance, both in the intricacy of the error and with respect to possible consequences of the error for the system. In contrast to the bacterium, a cognitive system can have a predictive model that is inconsistent with its current situation, because it can extrapolate from situations it (or its ancestors) have already encountered. Consequently, when its models fail to meet expectations, it can revise and adjust those models based on experience. It is capable of learning, while a bacterium can only develop new models through evolution by natural selection. In addition, a cognitive agent is also capable of taking open-endedly more sophisticated measures to reduce further error (e.g., Kiverstein and Sims, 2021).

It is worth mentioning at this point that the concept of autopoiesis was originally developed in the context of cognitive systems, and its central notion of operational closure (which corresponds to organizational closure as used in our argument) applies to the very definition of (embodied) cognition (Maturana and Varela, 1980; Varela et al., 1991; Weber and Varela, 2002; Thompson, 2007). This view, also, is based on an entangled and mutually supportive trialectic between perception, action, and what is called structural coupling between the two (a non-representational form of cognitive processing). This suggests an account of how coherent cognitive processes (just like natural agency) can arise from the adaptive dynamics of opponent processing. It is an intriguing possibility, whose detailed exploration, unfortunately, goes beyond the scope of this article.

Even more speculative, but equally plausible, is the idea that true intentionality, awareness, and consciousness arise through the emergence of yet higher levels of trialectic dynamics. Their evolution would be associated with ever-more intricate internal predictive models that must, at some point, incorporate accurate models of other living systems and the cognizing organism itself, if it is to deal with complex ecological and social contexts. Although this is still far from an actual model of the evolution of consciousness (and does not provide a definition of what consciousness or subjective awareness actually is), it opens up new and potentially fruitful avenues to develop such accounts.

In any case, our main conclusion is the following: *it is a mistake to regard cognition and consciousness as some complicated forms of computation*. Instead, they are elaborations on basic natural agency which, in turn, is fundamentally based on relevance realization. In other words, if our argument is valid, agency, cognition, and consciousness are related in a very profound manner that has not yet been widely recognized (but see Sims, 2021; Mitchell, 2023). They are all ways by which organisms come to know the world (Roli et al., 2022). Although we disagree with Humberto Maturana that all organisms are cognitive agents, we *do* think it is correct to say that “to live is to know” (Maturana, 1988). At the very heart of this process is the ability to pick out what is relevant—to delimit an arena in a large world. This is not a formalizable or algorithmic process. It *is* the process of formalizing the world in Hilbert’s sense of turning ill-defined problems into well-defined ones (as already discussed by Savage, 1954). We do not create meaning through computation. We generate meaning through living and acting, which is how we get a grip on our reality.^20^

## 8 Conclusion

Aristotle, in “De Anima,” considered the soul as the distinguishing principle of living systems—their characteristic formal cause that sets them apart from the non-living (Lennox, 2000, 2021; Shields, 2020). This conception is entirely naturalistic: Aristotle’s “soul” (unlike Plato’s) is neither immortal nor transcendental. Instead, it is immanent in the peculiar organization (form) of living matter (Louie, 2013; Hofmeyr, 2018, 2021). In his “Nicomachean Ethics,” Aristotle separates living beings into three discrete categories, according to the structural complexity of their animating principle: (1) All living beings have “nutritive” vital functions, which are required to keep themselves alive, to grow and reproduce. (2) In addition, animals’ souls have an aspect that Aristotle called “sensitive,” associated with their ability to move around and actively perceive the world. (3) Finally, he ascribed “rational” capabilities to humans alone: the capacity of deliberative imagination, and the ability to make rational choices (Shields, 2020). One needs not practice those capabilities to be human, but having them is what demarcates us from all other living beings.^21^

Our argument is similar in spirit to Aristotle’s, but updated for our current age and more gradual in nature. The animating principle of living systems is now fully naturalized, without reducing it completely to the fundamental laws of physics. We can describe it as the peculiar hierarchical and self-referential organization of the processes involved in *autopoiesis* (see section “4 Biological organization and natural agency”), *anticipation* (see section “5 Basic biological anticipation”), and *adaptation* (see section “6 Affordance, goal, and action”; summarized in Figure 6). What distinguishes living from non-living systems is the way in which these physico-chemical processes interrelate, how they maintain and build constraints on their dynamics upon existing constraints through physical work enabled by free energy gradients in the local environment. The resulting co-emergent and co-constructive dynamics are entirely compatible with the known laws of physics (and what we know about far-from-equilibrium thermodynamics in particular), but are ultimately not explainable by or reducible to those laws alone.

Instead, the dynamics of living systems are radically contingent and (at least to some degree) generated from within their organization itself. This conveys a certain autonomy to the organism, which is not due to a lack of causal determination, and is not primarily driven by randomness. But neither is it entirely determined by immediate and automated reactions to environmental triggers. Rather, organismic autonomy resides in biological organization, represented by a trialectic interplay of subprocesses—which is collectively impredicative, continuous, and concurrent—each dynamic aspect of the process constantly requiring the other two to be present at all times for its continued existence. This kind of dynamic and emergent self-constraint is what imbues a living system with agency: the ability to self-manufacture, to set intrinsic goals and pursue them through the choice of appropriate action, and to explore and exploit one’s arena through dynamics that emanate from *within* the organism’s own organization. This, in a nutshell, summarizes the account of life we present here: *agential emergentism* (Walsh, 2013, 2015; see section “2 Agential emergentism”). It shows parallels to enactivism in cognitive research, with its conception of life as *adaptive sense-making* (Varela et al., 1991; Di Paolo, 2005; Thompson, 2007; Di Paolo et al., 2017). Seen from this perspective, *relevance realization offers itself as the unifying core activity that allows agents to delimit and thereby enact their arena*, the part of their large world that matters to them, that enables them to survive and thrive.

Agential emergentism implies that mechanistic explanations are not sufficient to explain all the phenomena of life or its evolution. It accepts that the behavior of an autonomous living agent is characterized by a kind of finality, and therefore calls for some kind of teleological explanation. On Rosen’s account, the final cause of the organism is simply the same as the sum total of its efficient causes: the purpose of an autopoietic system is to manufacture *itself* (Rosen, 1991; Louie, 2009, 2013, 2017a). Or in Aristotle’s original terms: “the being of living beings is to live.” Hofmeyr (2018, 2021) refines this account and makes it compatible with the Aristotelian distinction between efficient and formal causes, the latter describing the peculiar functional form which the efficient and material causes of the organism take to achieve organizational closure and continuity. As we have shown in section “4 Biological organization and natural agency,” this enables the organism to set its own intrinsic goals, which legitimizes us to talk about the purposes and aims of a living being, not only *as if* it had agency (Dennett, 1987; Okasha, 2018), but in full acknowledgment that *it actually does so* (Walsh, 2015; Mitchell, 2023; Jaeger, 2024b).

To better understand the kind of teleological explanation our account requires—and why it is perfectly naturalistic, and thus scientific—we must contrast it to the causal explanations familiar to scientists today (Walsh D., 2012; Walsh, 2015). While causal explanations account for *how* an effect is generated by its immediately preceding causes, naturalistic teleological explanations account for *why* an organism acts to attain a certain goal. The two are complementary—not the same kind of explanation at all.

Let us emphasize that naturalistic teleological explanation suffers from none of the difficulties usually attributed to such accounts. First, it does not require that effects be produced by non-actual causes, in particular, that future states causally generate present ones. As argued in section “5 Basic biological anticipation,” anticipation means “pulling the future into the present” through internal predictive models (Louie, 2012, 2017b; Rosen, 2012). These models, and the expectations they represent, are fully actualized at the current moment in time. Second, naturalistic teleological explanation does not presuppose intentionality or cognitive capabilities in organisms that have none. Predictive internal models can be based on simple evolved habituation. Finally, naturalistic teleological explanation does not have a problem with normativity, since our account naturalizes norms for any living being. Indeed, we *define* agency as the observable natural ability of a living system to initiate actions according to its own intrinsic norms (Moreno and Mossio, 2015; Djedovic, 2020). This ability is a direct and natural consequence of autopoietic, self-manufacturing organization.^22^ In summary, the kind of teleological explanation we are willing to accept is completely legitimate as a scientific explanation, and it is very precisely circumscribed. It encompasses teleological descriptions of organismic behavior, but explicitly excludes global teleology of the kind that postulates a target state of evolution (or the universe) as a whole (a so-called *omega point*).

Actually, opposition to any kind of preset evolutionary target state is a central hallmark of our view. In contrast to globally teleological or, indeed, strongly deterministic mechanistic or (pan)computationalist approaches, our agential emergentist perspective is radically *open* regarding the behavior of organisms and the future of evolution. Both are considered not only *unpredictable* (see Rosen’s claims about the lack of computability in section “4 Biological organization and natural agency”) but, more strongly, fundamentally *not prestatable* (see Kauffman’s notions of radical emergence and the adjacent possible in section “6 Affordance, goal, and action”). Life cannot be completely *formalized*. There is no predefined space of possibilities, nor is there a clear beginning or end to biological innovation and diversification.^23^

To live, to evolve, means to be engaged in infinite play (Carse, 1986). Infinite play means constantly changing the rules of the game. The evolving universe cannot be captured by a fixed set of elements or properties. This is why algorithms cannot predict radical emergence. This should come as no surprise, since we cannot even predict all possible theorems in sufficiently complex *formal* systems (Longo, 2011). So why should it be feasible in the context of the natural world? The space of its possibilities—the configuration space of the universe—is constantly co-evolving and expanding with its actual state. It is a large world we live in, not a small one, precisely *because* we are fragile and limited living beings. The possibilities inherent in our world are indefinite—potentially infinite. And we have a say in what is happening: as agents, we co-construct our arena (and thus our opportunities) as we live our lives and evolve (see also Lewontin, 1983).

This co-constructive dynamic enables the emergence of further higher-level organization (see section “7 To live is to know”). Unlike Aristotle, we do not subdivide the domain of the living into a specific number of discrete categories (or subsystems). Our approach is more gradual, processual, piecemeal, and open-ended. While the first three levels of living organization (autopoiesis, anticipation, and adaptation) arise directly at the origin of life, additional levels of dynamic organization emerge later as major transitions during the course of evolution (Maynard Smith and Szathmáry, 1995; Deacon, 2011; Szathmáry, 2015). Interesting candidates for such emergent levels are provided by animal cognition and the phenomenon of consciousness in humans and other highly complexified animals (see section “7 To live is to know”).^24^ This suggests that natural agency, cognition, and consciousness may have evolved along a common theme, each a successively more complex elaboration on its predecessors. At the heart of this emergent evolutionary process lies *relevance realization*.

Our evolutionary account of relevance realization states that all organisms—from the simplest bacteria to the most sophisticated humans—are able to realize what is relevant in their experienced environment, to delimit their arena. In other words, organisms (through their self-manufacturing and adaptive organization) *actualize* the process of relevance realization. We have outlined why this process lies at the core of natural agency, cognition, and consciousness. We have also argued that this process cannot be algorithmic or computational in nature. Limited beings in a large world must first *define* their problems before they can solve them by rule-based inference. This is what it means for an organism to come to know its world (Roli et al., 2022). Relevance realization is not a formalizable process, since it *is* the process of formalization, the process of turning ill-defined problems into well-defined ones. This process is never finished. Instead, it is groundless and non-dual—neither syntactic or semantic only (Meling, 2021). Only living beings can perform it, since it requires autopoiesis, anticipation, and adaptation. Algorithms, in stark contrast, never even encounter the problem of relevance, since they exist in perfectly well-defined small worlds, where there is only one possible frame and choosing a perspective is never an option (see section “1 Introduction”).

It is the integrated, multi-scale process of adaptation—physiological, behavioral, and evolutionary—that provides the means by which relevance can be realized in a non-algorithmic manner (see section “7 To live is to know”). What these adaptive processes have in common is that different strategies—not always compatible, and sometimes even contradicting each other—are played against each other. This kind of opponent processing is the fundamental principle underlying relevance realization (see section “Introduction”; Vervaeke et al., 2012; Andersen et al., 2022). The performance of each strategy is evaluated, either reflexively by the organism itself, or through the intergenerational consequences of its struggle for survival and reproductive success. This results in a dynamic, flexible, and opportunist deployment of a diverse range of strategies, with the ultimate outcome of an ameliorated fit between agent and arena. Such adaptive dynamics are neither internally coherent, logical, or rational by default, nor do they require a well-defined (algorithmic or heuristic) approach to problem-solving or optimization (cf. section “3 Relevance realization”). When an organism successfully realizes what is relevant to itself, it always builds on its previous idiosyncratic and contingent history and experience. This is the only way a limited being can make sense of a large world.

In this sense, agential emergentism is closely aligned with the attempt of explaining relevance realization in terms of predictive processing (Andersen et al., 2022). Both see the solution to the problem of relevance in terms of evolutionary, meliorative, and contingent adaptive processes. However, our argument goes further than that. Predictive processing (like any Bayesian approach) is limited in the following two ways. First, it is a formal methodology that does not help us understand how the relevant variables to be included in an internal predictive model are chosen in the first place. As a way around this problem, it is often assumed that the brain somehow monitors a fixed set of perception channels, with relevance ascribed to those inputs that show a characteristic dynamic of error reduction in their predictions (Andersen et al., 2022). However, this begs the question: how does a limited being in a large world establish those channels? Second, Bayesianism assumes prior probabilities on expectations for which no justification is given. The argument here is that the adaptive process, given enough time, converges to a set of posterior probabilities which are independent of the initial priors. However, it is legitimate to doubt that this assumption applies without further justification in the context of the adaptive behavior and evolution of organisms. In general, the structure of the adjacent possible is constructive and divergent, and we should not expect the kind of convergence of posterior probabilities that Bayesians presuppose. Life rarely seems to have time to settle into a steady state before moving on to the next challenge.

While predictive processing keeps an open mind toward aspects of large worlds that cannot be formalized, strongly (pan)computationalist approaches to agency and cognition (see, for example, Baluška and Levin, 2016; Levin, 2021; Bongard and Levin, 2023) fail to acknowledge or address the basic insight that relevance realization *cannot* be of an algorithmic nature. Basically, these approaches only work within small worlds, where (as we have established here) there is no problem of relevance. This fundamentally limits their applicability and usefulness in the large world of actual organismic experience. While computational explanations (such as those based on predictive processing) can be effective, and there is little doubt that they have led to impressive empirical success, they fail to be properly grounded in light of the deeper philosophical issues discussed in this article. In contrast, our agential approach does not require any unrealistic oversimplifying assumptions about the nature of reality. While still allowing for computationalist approaches as a part of its wider outlook (treating them explicitly as approximations or emulations of the physical processes being studied by simulation), it provides several advantages over (pan)computationalism as a philosophical stance in terms of its explanatory power and the range of questions it is able to address.

In particular, evolutionary relevance realization allows us to ground the economic account (Vervaeke et al., 2012) in an organismic context beyond human cognition. Opponent processing and the question of when and how to commit to different strategies (see section “3 Relevance realization”) still lie at the core of realizing relevance, but they are now embedded in the basic processes of physiological, behavioral, and evolutionary adaptation. Relevance realization occurs in very simple organisms without intentionality or cognitive capacities whose internal predictive models and action repertoires are evolved by natural selection. A bacterium acting to enhance its chances of survival and reproduction by modulating its tumbling frequencies to avoid toxins and to obtain nutrition is no less realizing relevance than a human being deliberately contemplating whether to explore or exploit their opportunities in a given situation. The difference between the two situations is merely one of complexity, as Aristotle already recognized, and—if our considerations are correct—there is a gradual continuity and fundamental connection underlying these very distinct phenomena.

This sheds important light on current debates about rationality in humans (Riedl and Vervaeke, 2022). Specifically, it lends strong credence to the notions of embodied heuristics (Gigerenzer, 2021) and embodied bounded rationality (Mastrogiorgio and Petracca, 2016; Gallese et al., 2020; Petracca, 2021; Petracca and Grayot, 2023). Rationality, in the broadest sense of the term, can be defined as “knowing how to do the appropriate thing” in a particular situation (Riedl and Vervaeke, 2022, and references therein). Contra Aristotle, we do not consider humans as a uniquely “rational animal.” “Knowing how to do the appropriate thing” is not limited to human beings. Instead, human rationality can be seen as a powerful cognitive tool that gradually evolved from less intricate forms of natural agency to solve particularly multifaceted problems situated in a particularly complex natural and social environment. While problem-solving in a large world relies on abductive reasoning, it alone is not sufficient (cf. section “3 Relevance realization”; Roli et al., 2022). The “embodied” part of rationality means that, in order to solve problems through logical inference, we must first turn ill-defined large-world problems into well-defined small-world ones. And this is what relevance realization does, not only in humans, but in all living organisms: it generates the predictive hypotheses and models we need to be able to engage in abduction.

This primal activity of relevance realization is not “rational” in the sense of “logical.” Instead, it is based on the unprestatable and unformalizable adaptive dynamic of opponent processing, which is required to select those strategies that are likely to work best in a given situation. Therefore, and this should be obvious by now, to be rational in the sense of “doing the appropriate thing” does not always imply doing the most logical thing. Rather, it means doing the appropriate thing in a much more basic and pragmatic sense: initiating that sequence of actions most productive toward the attainment of our current goal, given both the affordance landscape of our arena and the repertoire of actions (and, in the case of humans, the cognitive resources) available to us. This is the extension of an argument made in Riedl and Vervaeke (2022) to non-human agents: human embodied rationality is a particularly complex form of a natural agency that is present in all living beings. It grows out of one of the most fundamental aspects of life: the necessity to make sense of a large world in order to survive and thrive.

Computational rationality—with its view that rational reasoning means formal optimization under certain cognitive resource constraints—is no longer sufficient as a basis for general intelligence. Instead, it is just one of many facets that contributes to our ability to understand the world (Roitblat, 2020; Roli et al., 2022). One of us has outlined in detail elsewhere what this means for current discussions about artificial “intelligence” (Jaeger, 2024a). The view that intelligence equals some kind of computational optimization is no longer tenable. It does not help us make sense of a large world. Therefore, claims that the study of intelligence is converging onto computational rationality as its ultimate foundation (see, for instance, Gershman et al., 2015) are not only premature, but outright misguided. Quite the opposite: we have shown here that the basic foundation of natural agency and cognition, and therefore of anything we could reasonably call “intelligence,” cannot be computational at all because it cannot be completely formalized. The dream of generating purely algorithmic systems able to think and act like human beings is and remains a pipe dream, because purely symbolic machines exist in small worlds, in which there is no problem of relevance to be solved.

As a final point, we would like to highlight the parallels between our approach and some branches of (meta)ethical philosophy. Cantwell Smith (2019) points out that genuine care is not possible in artificial algorithmic systems, because it depends on inhabiting a (large) world and being responsible for the inevitably partial ways in which we register it, while committing to such partial registrations and “going to bat” for them, sometimes at great cost. Similarly (and more famously), Frankfurt (1988, 2004, 2006) argues that any moral consideration must be evaluated against the background of what we care about as human beings. This background, as he demonstrates, cannot be based on pre-established ethical principles without causing an infinite regress. The problem Frankfurt describes is strikingly similar to that of relevance realization. What we care about, of course, is what is relevant to us. Only if we care about something can we choose the appropriate kind of action. Only by acting in the world can we get to know it. This is the very foundation of our knowledge and our morals. It is also what connects us to the rest of the living world. Life is meaningful and precious that way. No machine will ever understand that.

## Data availability statement

The original contributions presented in this study are included in this article/supplementary material, further inquiries can be directed to the corresponding author.

## Author contributions

JJ: Conceptualization, Investigation, Writing – original draft, Writing – review & editing. AR: Conceptualization, Investigation, Writing – original draft, Writing – review & editing. AD: Conceptualization, Investigation, Writing – original draft, Writing – review & editing. JV: Conceptualization, Investigation, Writing – original draft, Writing – review & editing. DW: Conceptualization, Investigation, Writing – original draft, Writing – review & editing.

## Footnotes

1 See also: https://aeon.co/essays/are-humans-really-blind-to-the-gorilla-on-the-basketball-court.

2 Relevance realization should not be confused with relevance theory (Sperber and Wilson, 1986, 1996; Oaksford, 1995; Wilson and Niv, 2012; Wilson and Sperber, 2012; Wilson, 2017; Sumers et al., 2022), which deals with the interpretation of utterances in human communication. While the latter applies only in the domain of language, the former is embedded and embodied by the active process of an organism making sense of its large world.

3 “Dialectic” is a term heavily burdened with different (often metaphysical and even political) interpretations. Here, we define it to simply mean the dynamic of two (or more) interrelated processes whose interactions do not merely feedback on each other, but are reciprocally constituting. In other words, the interacting processes support each other’s very existence by mutual co-construction.

4 As is the case for all analogies, this one also has its limitations. One important way in which “relevance” and “fitness” differ is that the former is a genuine property of the organism in its environment (the agent in its arena, see below), while the latter is only a property of an organism in a very attenuated sense: “fitness” is an abstraction from all the things an organism can actually do to get by, a sort of projection of its prospects. This caveat is important to keep in mind when considering analogous properties of “relevance” and “fitness” in what follows.

5 The distinction between agential emergentism and computationalism is, of course, much richer and deeper than just considering agency and cognition the same or not. We will elaborate on this in the following sections.

6 Pancomputationalists often call it a “principle,” claiming that it is (or can be) empirically verified, when in fact it remains nothing but philosophical conjecture (see, for example, Deutsch, 1997).

7 Of course, assessing outcomes and improving performance based on this kind of assessment is the fundamental principle of reinforcement learning in computer science (Sutton and Barto, 1998). But this only happens in a strictly small-world context, as the target function of the learning process *must* be provided externally, and the algorithm is not intrinsically motivated by the reinforcement.

8 To be more precise: all existing physical theories first establish a predefined phase or state space with a well-defined number of dimensions, which may be infinite as is the case for Hilbert spaces in quantum dynamics. All of these abstract spaces, even the infinite-dimensional ones, are mathematically prestatable by a finite number of axioms, due to the presence of symmetries. See Kauffman (2000), and also the lecture by Longo “Space and time in the foundations of mathematics,” available at: https://www.di.ens.fr/users/longo/files/PhilosophyAndCognition/space-time.pdf.

9 An algorithm can be provided with several predefined frames, with appropriate rules to select between them. This simply constitutes a larger predefined (meta)frame, but not the ability to truly frame one’s own problems.

10 Note that we use “symbolic processing” in the broad sense of “processing something that stands for something else by reason of a relation” (Pattee and Rączaszek-Leonardi, 2012). This is not the same as the more specific use of the term “symbolic” that characterizes an algorithm based on high-level (human-readable) problem representations, in contrast to subsymbolic (e.g., connectionist) methods that are used in artificial intelligence research.

11 There is a debate to be had whether Rosen muddles up Aristotelian efficient and formal causes. We will revisit this problem when discussing Hofmeyr’s elaboration of Rosen’s model below. In the meantime, to avoid confusion, we will stick with Rosen’s own terminology in our description of his work.

12 The term “collective impredicativity” has caused some controversy in the past, especially regarding Rosen’s claim that living systems are not computable. In particular, Mossio et al. (2009) have shown that Rosen’s (M,R)-system *can* be simulated using the formalism of λ-calculus. But this does not invalidate our argument or use of the term here. The difference between *recursion* (what the λ-simulation achieves) and *impredicativity* in a material sense (what the organism *does*) lies in Hofmeyr’s starting problem, which only arises if we consider recursion *in a physically embodied context*, as a process of explicit material co-construction, not just symbolic recursive calculation. It is this co-constructive aspect of a physical living system that we take to be analogous to the notion of a collectively impredicative set of definitions in mathematics. At the same time, we agree with Mossio et al. (2009) that Rosen’s claim seems to be missing the point: computability is not the issue, but formalizability is (see below).

13 Actually, the situation is a bit more subtle than that, as argued by Egbert and Barandiaran (2011). Using a minimalistic metabolic model of self-manufacture and agency, they show that between the dynamic regimes of “being (meta)stably alive” and “being dead” lies a precarious zone in which the system is slowly dying, but still alive for a limited amount of time.

14 The flipside of this coin is social/extended cognition, which also applies to organismic agency in the sense that other living beings, and inanimate objects in the agent’s arena, contribute in an essential way to the agency/cognition of a situated individual (e.g., Di Paolo, 2009). If you are deprived of education, you cannot act or think the same way as if you were educated. Computers, these days, allow us to do all kinds of things we could not do and to think in ways which would be impossible without them. Similar collective forms of agency/cognition abound. Based on this, some authors have suggested that living agents, together with the inanimate objects they interact with, can form a higher-level autonomous system (see, for example, Zebrowski and McGraw, 2021). We find this idea fascinating, but it does not change the fact that the ultimate source and precondition of agency in these extended and situated systems is still the involvement of a living agent—without life, no relevance realization, whether extended or not.

15 The doctrine of reinforcement learning (Sutton and Barto, 1998) relies on machine “wanting” in the sense of doling out “rewards” and “punishments” to an algorithm for progressing toward a predefined goal, but the use of these terms is obviously metaphorical, as the target (as always in a machine context) is imposed externally (see text footnote 4). In stark contrast, the intrinsic motivation of an organism must ultimately be rooted in its continuous drive to persist in a challenging environment. The tedium and futility of any kind of true immortality is philosophically pondered in Bernard Williams’ classical essay “The Makropulos Case” (Williams, 1973).

16 An alternative explanation can be provided in terms of the “free-energy principle” (Friston, 2010, 2013; Kiverstein and Sims, 2021), which defines a set of minimal principles of organization for systems capable of adapting to their environments in terms of their capacity to minimize surprisal (abstracted as “free energy”), i.e., to seek out a path of least resistance or improbability. This can be interpreted as a simple instantiation of anticipatory behavior. However, this explanation remains embedded in an algorithmic (small-world) framework, as we discuss in section “8 Conclusion.”

17 Seen through the lens of embodied reinforcement learning, this seems to imply a simple grounding in the dopaminergic system (a mapping from perception to reward signal, which represents relevance; see, for example, Gershman et al., 2015). But, once again, this leaves open the question where goals and affordances come from in the first place, and how their relation to the reward system originated and subsequently adapted through evolution.

18 In this sense, our account resembles Peter Corning’s account of “holistic Darwinism” and “synergistic selection” (Corning, 1981, 2005, 2018), which focuses on the emergence of energy-capturing mechanisms during evolution, rather than the leveraging of affordances.

19 As an interesting analogy, consider the acquisition of expert intuition. “In the mind of the novice, there are many possibilities; in the mind of the master, only one.” This is based on a long process of training, leading to the kind of local adaptation to a specific part of reality that allows the expert to simplify (i.e., render *less* intricate) the problems at hand.

20 This parallels Joseph Weizenbaum’s prescient distinction between calculating and judging (Weizenbaum, 1976), which is itself based on earlier differentiations, e.g., Pascal’s “spirit of calculation” versus his “spirit of finesse.” In fact, we could say that pretty much all post-Cartesian attempts at phenomenology corroborate this basic dichotomy, but this foundation of our philosophy has been lost through (pan)computationalism over the past few decades.

21 In light of what is expressed in this paragraph, it is helpful (and not at all inaccurate although clearly anachronistic) to think of Aristotelian souls, not as entities, but as systems: the nutritive system, the locomotor system, the cognitive system. This is reflected in what he says about the dependence of one system on another, about their sharing of material parts, and about their vastly different realizations in different organisms.

22 The capability to stay alive, therefore, is the enabling prerequisite—the most essential lower bound—for getting to know one’s world (Roli et al., 2022). The connection between this lower bound of normativity and other varieties of normativity is, at the present time, not fully developed or understood (Djedovic, 2020).

23 The only known exception to this rule being the origin of life, of course.

24 There is also the possibility for entirely different branches of levels of living organization separate from cognition and consciousness. For example, immune systems might represent an entirely separate type of living organization, as might higher-order dynamics of eusocial insect colonies, or other forms of close mutualistic interactions. All these forms are elaborate, non-cognitive forms of relevance realization. Whether they are also based on some kind of triadic co-constructive dynamics remains to be investigated.

## References

[B1] Andersen B. P. Miller M. Vervaeke J. (2022). Predictive processing and relevance realization: exploring convergent solutions to the frame problem. *Phenom. Cogn. Sci.* 10.1007/s11097-022-09850-6

[B2] Anderson P. W. (1972). More is different. *Science* 177 393–396. 10.1126/science.177.4047.393 17796623

[B3] Andrews M. (2021). The math is not the territory: navigating the free energy principle. *Biol. Philos.* 36:30. 10.1007/s10539-021-09807-0

[B4] Arthur W. B. (1994). Inductive reasoning and bounded rationality. *Am. Econ. Rev.* 84 406–411.

[B5] Baker M. D. Stock J. B. (2007). Signal transduction: networks and integrated circuits in bacterial cognition. *Curr. Biol.* 17 R1021–R1024. 10.1016/j.cub.2007.10.011 18054766

[B6] Baluška F. Levin M. (2016). On having no head: cognition throughout biological systems. *Front. Psychol.* 7:902. 10.3389/fpsyg.2016.00902 27445884 PMC4914563

[B7] Barandiaran X. E. Di Paolo E. Rohde M. (2009). Defining agency: individuality, normativity, asymmetry, and spatio-temporality in action. *Adapt. Behav.* 17 367–386. 10.1177/1059712309343819

[B8] Barandiaran X. Moreno A. (2008). On the nature of neural information: a critique of the received view 50 years later. *Neurocomputing* 71 681–692. 10.1016/j.neucom.2007.09.014

[B9] Barbieri M. (2015). *Code Biology: a New Science of Life.* Cham, CH: Springer Intl. Publishing. 10.1007/978-3-319-14535-8

[B10] Bickhard M. H. (2000). Autonomy, function, and representation. *Commun. Cogn. Artif. Intell.* 17 111–131.

[B11] Birch J. Ginsburg S. Jablonka E. (2020). Unlimited associative learning and the origins of consciousness: a primer and some predictions. *Biol. Philos.* 35:56. 10.1007/s10539-020-09772-0 33597791 PMC7116763

[B12] Bongard J. Levin M. (2023). There’s plenty of room right here: biological systems as evolved, overloaded, multi-scale machines. *Biomimetics* 8:110. 10.3390/biomimetics8010110 36975340 PMC10046700

[B13] Bradie M. (1986). Assessing evolutionary psychology. *Biol. Philos.* 1 401–459. 10.1007/bf00140962

[B14] Bradie M. Harms W. (2023). “Evolutionary epistemology,” in *The Stanford Encyclopedia of Philosophy (Spring 2023)*, eds Zalta E. N. Nodelma U. (Stanford, CA: Metaphysics Research Lab, Stanford University).

[B15] Brighton H. (2018). Rationality without optimality: bounded and ecological rationality from a Marrian perspective. *PsyArXiv [Preprint]* 10.31234/osf.io/m2sz5

[B16] Campbell D. T. (1974). “Evolutionary epistemology,” in *The Philosophy of Karl R. Popper*, ed. Schilpp P. A. (La Salle, IL: Open Court), 231–257.

[B17] Cantwell Smith B. (2019). *The Promise of Artificial Intelligence: Reckoning and Judgment.* Cambridge, MA: The MIT Press. 10.7551/mitpress/12385.001.0001

[B18] Carney J. (2020). Thinking avant la lettre: a review of 4E cognition. *Evol. Stud. Imaginative Cult.* 4 77–90. 10.26613/esic.4.1.172 32457930 PMC7250653

[B19] Carse J. (1986). *Finite and Infinite Games: a Vision of Life As Play and Possibility.* New York, NY: Free Press.

[B20] Chemero A. (2003). An outline of a theory of affordances. *Ecol. Psychol.* 15 181–195. 10.1207/S15326969ECO1502_5

[B21] Church A. (1936). An unsolvable problem of elementary number theory. *Am. J. Math.* 58 345–363. 10.2307/2371045

[B22] Clark A. (2013). Whatever next? predictive brains, situated agents, and the future of cognitive science. *Behav. Brain Sci.* 36 181–204. 10.1017/S0140525X12000477 23663408

[B23] Clark A. (2015). *Surfing Uncertainty: Prediction, Action, and the Embodied Mind.* Oxford University Press: Oxford, UK. 10.1093/acprof:oso/9780190217013.001.0001

[B24] Colombo M. Wright C. (2021). First principles in the life sciences: the free-energy principle, organicism, and mechanism. *Synthese* 198 3463–3488. 10.1007/s11229-018-01932-w

[B25] Copeland B. J. (2020). “The church-turing thesis,” in *The Stanford Encyclopedia of Philosophy (Summer 2020)*, ed. Zalta E. N. (Stanford, CA: Metaphysics Research Lab, Stanford University).

[B26] Corcoran A. W. Pezzulo G. Hohwy J. (2020). From allostatic agents to counterfactual cognisers: active inference, biological regulation, and the origins of cognition. *Biol. Philos.* 35:32. 10.1007/s10539-020-09746-2

[B27] Corning P. A. (1981). *The Synergism Hypothesis: a Theory of Progressive Evolution.* New York, NY: McGraw-Hill.

[B28] Corning P. A. (2005). *Holistic Darwinism: Synergy, Cybernetics, and the Bioeconomics of Evolution.* Chicago, IL: University of Chicago Press. 10.7208/chicago/9780226116334.001.0001

[B29] Corning P. A. (2018). *Synergistic Selection: How Cooperation Has Shaped Evolution and the Rise of Humankind.* Singapore, SG: World Scientific. 10.1142/10732

[B30] Cornish-Bowden A. Cárdenas M. L. (2020). Contrasting theories of life: historical context, current theories. in search of an ideal theory. *BioSystems* 188:104063. 10.1016/j.biosystems.2019.104063 31715221

[B31] Cziko G. (1995). *Without Miracles: Universal Selection Theory and the Second Darwinian Revolution.* Cambridge, MA: The MIT Press.

[B32] Deacon T. W. (2011). *Incomplete Nature: How Mind Emerged from Matter*, 1st Edn. New York, NY: W. W. Norton & Company.

[B33] Deacon T. W. (2015). Steps to a science biosemiotics. *Green Lett. Stud. Ecocritic.* 19 293–311. 10.1080/14688417.2015.1072948

[B34] Dennett D. (1984). “Cognitive wheels: the frame problem of AI,” in *Minds, Machines and Evolution*, ed. Hookway C. (Cambridge: Cambridge University Press), 129–151.

[B35] Dennett D. C. (1987). *The Intentional Stance.* Cambridge, MA: The MIT Press.

[B36] Deutsch D. (1985). Quantum theory, the church–turing principle and the universal quantum computer. *Proc. Roy. Soc. Lond. A* 400 97–117. 10.1098/rspa.1985.0070

[B37] Deutsch D. (1997). *The Fabric of Reality.* New York, NY: Penguin Books.

[B38] Di Paolo E. Buhrmann T. Barandiaran X. (2017). *Sensorimotor Life: an Enactive Proposal.* Oxford: Oxford University Press. 10.1093/acprof:oso/9780198786849.001.0001

[B39] Di Paolo. (2005). Autopoiesis, adaptivity, teleology, agency. *Phenomenol. Cogn. Sci.* 4 429–452. 10.1007/s11097-005-9002-y

[B40] Di Paolo. (2009). Extended life. *Topoi* 28 9–21. 10.1007/s11245-008-9042-3

[B41] DiFrisco J. Mossio M. (2020). “Diachronic identity in complex life cycles: an organizational perspective,” in *Biological Identity: Perspectives from Metaphysics and the Philosophy of Biology*, eds Meincke A. S. Dupré J. (London: Routledge). 10.4324/9781351066389-10

[B42] Djedovic A. (2020). *From Life-like to Mind-like Explanation: Natural Agency and the Cognitive Sciences.* Ph.D. Thesis. Toronto: University of Toronto.

[B43] Dreyfus H. L. (1979). *What Computers Can’t Do: the Limits of Artificial Intelligence (Revised Edition; original: 1972).* New York, NY: HarperCollins.

[B44] Dreyfus H. L. (1992). *What Computers Still Can’t Do: a Critique of Artificial Reason.* Cambridge, MA: The MIT Press.

[B45] Durt C. Fuchs T. Tewes C. (2017). *Embodiment, Action, and Culture: Investigating the Constitution of the Shared World.* Cambridge, MA: The MIT Press. 10.7551/mitpress/9780262035552.001.0001

[B46] Egbert M. D. Barandiaran X. E. (2011). “Quantifying normative behaviour and precariousness in adaptive agency,” in *Advances in Artificial Life—Proceedings of the 11th European Conference on Artificial Life (ECAL)*, Vol. 11 eds Lenaerts T. Giacobini M. Bersini H. Bourgine P. Dorigo M. Doursa R. (Cambridge, MA: The MIT Press), 201–218.

[B47] Falcon A. (2023). “Aristotle on causality,” in *The Stanford Encyclopedia of Philosophy (Spring 2023)*, eds Zalta E. N. Nodelman U. (Stanford, CA: Metaphysics Research Lab, Stanford University).

[B48] Favareau D. (2010). *Essential Readings in Biosemiotics: Anthology and Commentary.* Dordrecht, NL: Springer Netherlands. 10.1007/978-1-4020-9650-1

[B49] Felin T. Felin M. (2019). “Seeking rationality: $500 bills and perceptual obviousness,” in *The Routledge Handbook of Bounded Rationality*, ed. Viale R. (London: Routledge), 103–119. 10.4324/9781315658353-6

[B50] Felin T. Kauffman S. (2019). “The search function and evolutionary novelty,” in *New Developments in Evolutionary Innovation: Novelty Creation in a Serendipitous Economy*, eds Cattani G. Mastrogiorgio M. (Oxford: Oxford University Press), 113–143. 10.1093/oso/9780198837091.003.0007

[B51] Felin T. Koenderink J. (2022). A generative view of rationality and growing awareness. *Front. Psychol.* 13:807261. 10.3389/fpsyg.2022.807261 35465538 PMC9021390

[B52] Felin T. Koenderink J. Krueger J. I. (2017). Rationality, perception, and the all-seeing eye. *Psychon. Bull. Rev.* 24 1040–1059. 10.3758/s13423-016-1198-z 27928763 PMC5570804

[B53] Feyerabend P. (1975). *Against Method.* London: New Left Books.

[B54] Frankfurt H. G. (1988). *The Importance of What We Care About: Philosophical Essays.* Cambridge: Cambridge University Press. 10.1017/CBO9780511818172

[B55] Frankfurt H. G. (2004). *The Reasons of Love.* Princeton, NJ: Princeton University Press.

[B56] Frankfurt H. G. (2006). *Taking Ourselves Seriously and Getting It Right.* Stanford, CA: Stanford University Press.

[B57] Frankl V. E. (1946). *Trotzdem Ja zum Leben sagen—Ein Psychologe erlebt das Konzentrationslager.* Vienna, AT: Verlag für Jugend und Volk.

[B58] Frankl V. E. (2020). *Yes to Life: in Spite of Everything.* Boston, MA: Beacon Press.

[B59] Friston K. (2010). The free-energy principle: a unified brain theory? *Nat. Rev. Neurosci.* 11 127–138. 10.1038/nrn2787 20068583

[B60] Friston K. (2013). Life as we know it. *J. Roy. Soc. Interface* 10:20130475. 10.1098/rsif.2013.0475 23825119 PMC3730701

[B61] Friston K. Breakspear M. Deco G. (2012). Perception and self-organized instability. *Front. Comp. Neurosci.* 6:44. 10.3389/fncom.2012.00044 22783185 PMC3390798

[B62] Fulda F. C. (2017). Natural agency: the case of bacterial cognition. *J. Am. Philos. Assoc.* 3 69–90. 10.1017/apa.2017.5

[B63] Gallese V. Mastrogiorgio A. Petracca E. Viale R. (2020). “Embodied bounded rationality,” in *Palgrave Handbook of Bounded Rationality*, ed. Viale R. (London: Palgrave Macmillan), 377–390. 10.4324/9781315658353-26

[B64] Gershman S. J. Horvitz E. J. Tenenbaum J. B. (2015). Computational rationality: a converging paradigm for intelligence in brains, minds, and machines. *Science* 349 273–278. 10.1126/science.aac6076 26185246

[B65] Gibson J. J. (1966). *The Senses Considered as Perceptual Systems.* London: Houghton Mifflin.

[B66] Gibson J. J. (1979). *The Ecological Approach to Visual Perception.* London: Houghton Mifflin.

[B67] Gigerenzer G. (2021). Embodied heuristics. *Front. Psychol.* 12:711289. 10.3389/fpsyg.2021.711289 34858251 PMC8631174

[B68] Gigerenzer G. Gaissmaier W. (2011). Heuristic decision making. *Ann. Rev. Psychol.* 62 451–482. 10.1146/annurev-psych-120709-145346 21126183

[B69] Haggard P. (2008). Human volition: towards a neuroscience of will. *Nat. Rev. Neurosci.* 9 934–946. 10.1038/nrn2497 19020512

[B70] Harrison D. Rorot W. Laukaityte U. (2022). Mind the matter: Active matter, soft robotics, and the making of bio-inspired artificial intelligence. *Front. Neurorobot.* 16:880724. 10.3389/fnbot.2022.880724 36620483 PMC9815774

[B71] Haugeland J. (1998). *Having Thought: Essays in the Metaphysics of Mind.* Cambridge, MA: Harvard University Press.

[B72] Heras-Escribano M. (2019). *The Philosophy of Affordances.* Cham, CH: Springer Intl. Publishing. 10.1007/978-3-319-98830-6

[B73] Hofmeyr J.-H. S. (2007). “The biochemical factory that autonomously fabricates itself: a systems biological view of the living cell,” in *Systems Biology - Philosophical Foundations*, eds Boogerd F. Bruggeman F. J. Hofmeyr J.-H. S. Westerhoff H. V. (Dordrecht, NL: Elsevier), 217–242. 10.1016/B978-044452085-2/50012-7

[B74] Hofmeyr J.-H. S. (2017). “Basic biological anticipation,” in *Handbook of Anticipation*, ed. Poli R. (Cham, CH: Springer Intl. Publishing), 1–15. 10.1007/978-3-319-31737-3_51-1

[B75] Hofmeyr J.-H. S. (2018). Causation, constructors and codes. *BioSystems* 164 121–127. 10.1016/j.biosystems.2017.09.008 28916462

[B76] Hofmeyr J.-H. S. (2021). A biochemically-realisable relational model of the self-manufacturing cell. *BioSystems* 207:104463. 10.1016/j.biosystems.2021.104463 34166730

[B77] Hofstadter D. R. Sander E. (2013). *Surfaces and Essences: Analogy as the Fuel and Fire of Thinking.* New York, NY: Basic Books.

[B78] Hohwy J. (2014). *The Predictive Mind.* Oxford: Oxford University Press. 10.1093/acprof:oso/9780199682737.001.0001

[B79] Jaeger J. (2019). “Dynamic structures in evo-devo: from morphogenetic fields to evolving organisms,” in *Perspectives on Evolutionary and Developmental Biology — Essays for Alessandro Minelli*, ed. Fusco G. (Padova, IT: Padova University Press), 335–356.

[B80] Jaeger J. (2024a). “Artificial intelligence is algorithmic mimicry: why artificial ‘agents’ are not (and won’t be) proper agents,” in *Neurons, Behavior, Data Analysis, and Theory*, 1–21. 10.51628/001c.94404

[B81] Jaeger J. (2024b). “The fourth perspective: evolution and organismal agency,” in *Organization in Biology*, ed. Mossio M. (Berlin: Springer International Publishing), 159–186. 10.1007/978-3-031-38968-9_8

[B82] Jaeger J. (2024c). “Ontogenesis, organization, and organismal agency,” in *Organismal Agency: Biological Concepts and Their Philosophical Foundations*, ed. Švorcová J. (Cham, CH: Springer Nature). 10.1007/978-3-031-53626-7_10

[B83] Jaeger J. Monk N. (2014). Bioattractors: dynamical systems theory and the evolution of regulatory processes. *J. Physiol.* 592 2267–2281. 10.1113/jphysiol.2014.272385 24882812 PMC4048087

[B84] Jaeger J. Irons D. Monk N. (2012). The inheritance of process: a dynamical systems approach: the inheritance of process. *J. Exp. Zool.* 318B 591–612. 10.1002/jez.b.22468 23060018

[B85] Jonas H. (1966). *The Phenomenon of Life: Toward a Philosophical Biology.* New York, NY: Harper & Row.

[B86] Juarrero A. (1999). *Dynamics in Action: Intentional Behavior as a Complex System.* Cambridge, MA: The MIT Press. 10.7551/mitpress/2528.001.0001

[B87] Juarrero A. (2023). *Context Changes Everything: How Constraints Create Coherence.* Cambridge, MA: The MIT Press. 10.7551/mitpress/14630.001.0001

[B88] Kauffman S. A. (1971). “Articulation of parts explanation in biology and the rational search for them,” in *PSA 1970*, Vol. 8 eds Buck R. C. Cohen R. S. (Boston, MA: Reidel), 257–272. 10.1007/978-94-010-3142-4_18

[B89] Kauffman S. A. (2000). *Investigations.* Oxford: Oxford University Press. 10.1093/oso/9780195121049.001.0001

[B90] Kiverstein J. Sims M. (2021). Is free-energy minimisation the mark of the cognitive? *Biol. Philos.* 36:25. 10.1007/s10539-021-09788-0

[B91] Kiverstein J. Miller M. Rietveld E. (2019). The feeling of grip: Novelty, error dynamics, and the predictive brain. *Synthese* 196 2847–2869. 10.1007/s11229-017-1583-9

[B92] Ladyman J. Wiesner K. (2020). *What Is a Complex System?.* New Haven, CT: Yale University Press. 10.12987/yale/9780300251104.001.0001

[B93] Lee J. G. McShea D. W. (2020). Operationalizing goal directedness: an empirical route to advancing a philosophical discussion. *PTPbiol* 12:5. 10.3998/ptpbio.16039257.0012.005 26400043

[B94] Lennox J. (2021). “Aristotle’s biology,” in *The Stanford Encyclopedia of Philosophy (Fall 2021)*, ed. Zalta E. N. (Stanford, CA: Metaphysics Research Lab, Stanford University).

[B95] Lennox J. G. (2000). *Aristotle’s Philosophy of Biology: Studies in the Origins of Life Science.* Cambridge: Cambridge University Press.

[B96] Letelier J.-C. Cárdenas M. L. Cornish-Bowden A. (2011). From L’Homme machine to metabolic closure: steps towards understanding life. *J. Theor. Biol.* 286 100–113. 10.1016/j.jtbi.2011.06.033 21763318

[B97] Levin M. (2021). Life, death, and self: fundamental questions of primitive cognition viewed through the lens of body plasticity and synthetic organisms. *Biochem. Biophys. Res. Commun.* 564 114–133. 10.1016/j.bbrc.2020.10.077 33162026

[B98] Lewontin R. (1983). The organism as the subject and object of evolution. *Scientia* 118 63–82.

[B99] Lloyd S. (2006). *Programming the Universe: a Quantum Computer Scientist Takes on the Cosmos.* New York, NY: Alfred A. Knopf.

[B100] Longo G. (2009). “Critique of computational reason in the natural sciences,” in *Advances in Computer Science and Engineering*, Vol. 3 eds Gelenbe E. Kahane J.-P. (Singapore, SG: World Scientific Publishing), 43–70. 10.1142/9781848162914_0003

[B101] Longo G. (2011). Reflections on concrete incompleteness. *Philos. Math.* 19 255–280. 10.1093/phimat/nkr016

[B102] Longo G. Paul T. (2011). “The mathematics of computing between logic and physics,” in *Computability in Context*, eds Cooper S. B. Sorbi A. (Singapore, SG: World Scientific Publishing), 243–273. 10.1142/9781848162778_0007

[B103] Lorenz K. (1977). *Behind the Mirror.* London: Methuen.

[B104] Louie A. H. (2009). *More Than Life Itself: a Synthetic Continuation in Relational Biology.* Frankfurt, DE: Ontos Verlag. 10.1515/9783110321944

[B105] Louie A. H. (2012). Anticipation in (M,R)-systems. *Intl. J. Gen. Syst.* 41 5–22. 10.1080/03081079.2011.622088

[B106] Louie A. H. (2013). *The Reflection of Life.* Berlin, DE: Springer. 10.1007/978-1-4614-6928-5

[B107] Louie A. H. (2017a). *Intangible Life.* Publishing: Cham: Springer Intl. 10.1007/978-3-319-65409-6

[B108] Louie A. H. (2017b). “Mathematical foundations of anticipatory systems,” in *Handbook of Anticipation*, ed. Poli R. (Cham CH: Springer Nature Switzerland), 937–964. 10.1007/978-3-319-91554-8_21

[B109] Louie A. H. Poli R. (2017). “Complex systems,” in *Handbook of Anticipation*, ed. Poli R. (Cham, CH: Springer Nature), 17–35. 10.1007/978-3-319-91554-8_3

[B110] Lyon P. (2006). The biogenic approach to cognition. *Cogn. Proc.* 7 11–29. 10.1007/s10339-005-0016-8 16628463

[B111] Lyon P. Keijzer F. Arendt D. Levin M. (2021). Reframing cognition: getting down to biological basics. *Philos. Trans. Roy. Soc. B* 376:20190750. 10.1098/rstb.2019.0750 33487107 PMC7935032

[B112] Mastrogiorgio A. Petracca E. (2016). “Embodying rationality,” in *Model-Based Reasoning in Science and Technology*, eds Magnani L. Casadio C. (Cham, CH: Springer Intl. Publishing), 219–237. 10.1007/978-3-319-38983-7_12

[B113] Maturana H. R. (1988). Reality: the search for objectivity or the quest for a compelling argument. *Irish. J. Psychol.* 9 25–82. 10.1080/03033910.1988.10557705

[B114] Maturana H. R. Varela F. J. (1980). *Autopoiesis and Cognition: the Realization of the Living.* Berlin, DE: Springer. 10.1007/978-94-009-8947-4

[B115] Maynard Smith J. Szathmáry E. (1995). *The Major Transitions in Evolution.* Oxford: Oxford University Press.

[B116] Mayr E. (1974). “Teleological and teleonomic, a new analysis,” in *Methodological and Historical Essays in the Natural and Social Sciences*, eds Cohen R. S. Wartofsky M. W. (Boston, MA: Reidel), 91–117. 10.1007/978-94-010-2128-9_6

[B117] Mayr E. (1982). *The Growth of Biological Thought: Diversity, Evolution, and Inheritance.* Cambridge, MA: Belknap Press: An Imprint of Harvard University Press.

[B118] Mayr E. (1992). The idea of teleology. *J. Hist. Ideas* 53 117–135. 10.2307/2709913

[B119] McCarthy J. Hayes P. J. (1969). “Some philosophical problems from the standpoint of artificial intelligence,” in *Machine Intelligence*, Vol. 4 eds Michie D. Meltzer B. (Edinburgh: Edinburgh University Press), 463–502.

[B120] McShea D. W. (2012). Upper-directed systems: a new approach to teleology in biology. *Biol. Philos.* 27 663–684. 10.1007/s10539-012-9326-2

[B121] McShea D. W. (2013). Machine wanting. *Stud. Hist. Philos. Sci. C Biol. Biomed. Sci.* 44 679–687. 10.1016/j.shpsc.2013.05.015 23792091

[B122] McShea D. W. (2016). Freedom and purpose in biology. *Stud. Hist. Philos. Sci. C Biol. Biomed. Sci.* 58 64–72. 10.1016/j.shpsc.2015.12.002 26777154

[B123] Meling D. (2021). Knowing groundlessness: an enactive approach to a shift from cognition to non-dual awareness. *Front. Psychol.* 12:697821. 10.3389/fpsyg.2021.697821 34421749 PMC8377755

[B124] Mitchell K. (2023). *Free Agents: How Evolution Gave Us Free Will.* Princeton, NJ: Princeton University Press. 10.1515/9780691226224

[B125] Mitchell M. (2009). *Complexity: a Guided Tour.* Oxford: Oxford University Press. 10.1093/oso/9780195124415.001.0001

[B126] Montévil M. Mossio M. (2015). Biological organisation as closure of constraints. *J. Theor. Biol.* 372 179–191. 10.1016/j.jtbi.2015.02.029 25752259

[B127] Montévil M. Mossio M. Pocheville A. Longo G. (2016). Theoretical principles for biology: variation. *Prog. Biophys. Mol. Biol.* 122 36–50. 10.1016/j.pbiomolbio.2016.08.005 27530930

[B128] Moreno A. Etxeberria A. (2005). Agency in Natural and Artificial Systems. *Artificial Life* 11 161–175. 10.1162/1064546053278919 15811225

[B129] Moreno A. Mossio M. (2015). *Biological Autonomy.* Dordrecht, NL: Springer Netherlands. 10.1007/978-94-017-9837-2

[B130] Moreno A. Umerez J. Ibañez J. (1997). Cognition and life: the autonomy of cognition. *Brain Cogn.* 34 107–129. 10.1006/brcg.1997.0909 9209758

[B131] Mossio M. (2024a). *Organization in Biology.* Cham, CH: Springer Intl. Publishing. 10.1007/978-3-031-38968-9

[B132] Mossio M. (2024b). “Introduction: organization as a scientific blind spot,” in *Organization in Biology*, ed. Mossio M. (Cham, CH: Springer Intl. Publishing), 1–22. 10.1007/978-3-031-38968-9_1

[B133] Mossio M. Bich L. (2017). What makes biological organisation teleological? *Synthese* 194 1089–1114. 10.1007/s11229-014-0594-z

[B134] Mossio M. Pontarotti G. (2020). Conserving functions across generations: heredity in light of biological organization. *Br. J. Philos. Sci.* 73:axz031. 10.1093/bjps/axz031 32691291

[B135] Mossio M. Longo G. Stewart J. (2009). A computable expression of closure to efficient causation. *J. Theor. Biol.* 257 489–498. 10.1016/j.jtbi.2008.12.012 19168079

[B136] Mossio M. Montévil M. Longo G. (2016). Theoretical principles for biology: organization. *Prog. Biophys. Mol. Biol.* 122 24–35. 10.1016/j.pbiomolbio.2016.07.005 27521451

[B137] Nagel E. Newman J. R. (2001). *Gödel’s Proof (Revised Edition).* New York, NY: NYU Press.

[B138] Newell A. Simon H. A. (1972). *Human Problem Solving.* Englewood Cliffs, NY: Prentice-Hall.

[B139] Newen A. de Bruin L. Gallagher S. (2018). *The Oxford Handbook of 4E Cognition.* Oxford: Oxford University Press. 10.1093/oxfordhb/9780198735410.001.0001

[B140] Nicholson D. J. (2013). Organisms ≠ machines. *Stud. Hist. Philos. Sci. C Biol. Biomed. Sci.* 44 669–678. 10.1016/j.shpsc.2013.05.014 23810470

[B141] Oaksford M. (1995). Information gain explains relevance which explains the selection task. *Cognition* 57 97–108. 10.1016/0010-0277(95)00671-K 7587019

[B142] Okasha S. (2018). *Agents and Goals in Evolution.* Oxford: Oxford University Press. 10.1093/oso/9780198815082.001.0001

[B143] Pattee H. H. (2001). The physics of symbols: bridging the epistemic cut. *BioSystems* 60 5–21. 10.1016/S0303-2647(01)00104-6 11325500

[B144] Pattee H. H. Rączaszek-Leonardi J. (2012). *Laws, Language and Life: Howard Pattee’s Classic Papers on the Physics of Symbols with Contemporary Commentary.* Dordrecht, NL: Springer Netherlands. 10.1007/978-94-007-5161-3

[B145] Petracca E. (2021). Embodying bounded rationality: from embodied bounded rationality to embodied rationality. *Front. Psychol.* 12:710607. 10.3389/fpsyg.2021.710607 34566788 PMC8459109

[B146] Petracca E. Grayot J. (2023). How can embodied cognition naturalize bounded rationality? *Synthese* 201:115. 10.1007/s11229-023-04124-3

[B147] Piaget J. (1967). *Biologie et Connaissance.* Paris, FR: Idées/Gallimard.

[B148] Polanyi M. (1968). Life’s irreducible structure. *Science* 160 1308–1312. 10.1126/science.160.3834.1308 5651890

[B149] Pontarotti G. (2024). “Organization and inheritance in twenty-first-century evolutionary biology,” in *Organization in Biology*, ed. Mossio M. (Cham, CH: Springer Intl. Publishing), 219–240. 10.1007/978-3-031-38968-9_10

[B150] Ratcliffe M. (2012). “There can be no cognitive science of dasein,” in *Heidegger and Cognitive Science*, eds Kiverstein J. Wheeler M. (London: Palgrave Macmillan), 135–156. 10.1007/978-1-137-00610-3_4

[B151] Riedl A. Vervaeke J. (2022). Rationality and relevance realization. synthese (forthcoming). *OSF [Preprint]* 10.31219/osf.io/vymwu

[B152] Roitblat H. L. (2020). *Algorithms Are Not Enough—Creating Artificial General Intelligence.* Cambridge, MA: The MIT Press. 10.7551/mitpress/11659.001.0001

[B153] Roli A. Jaeger J. Kauffman S. A. (2022). How organisms come to know the world: fundamental limits on artificial general intelligence. *Front. Ecol. Evol.* 9:806283. 10.3389/fevo.2021.806283

[B154] Rolla G. Figueiredo N. (2023). Bringing forth a world, literally. *Phenom. Cogn. Sci.* 22 931–953. 10.1007/s11097-021-09760-z

[B155] Rosen R. (1958a). A relational theory of biological systems. *Bull. Math. Biophys.* 20 245–260. 10.1007/BF02478302

[B156] Rosen R. (1958b). The representation of biological systems from the standpoint of the theory of categories. *Bull. Math. Biophys.* 20 317–341. 10.1007/BF02477890

[B157] Rosen R. (1959). A relational theory of biological systems II. *Bull. Math. Biophys.* 21 109–128. 10.1007/BF02476354

[B158] Rosen R. (1972). “Some relational cell models: the metabolism-repair systems,” in *Foundations of Mathematical Biology*, Vol. II ed. Rosen R. (New York, NY: Academic Press), 217–253. 10.1016/B978-0-12-597202-4.50011-6

[B159] Rosen R. (1991). *Life Itself: a Comprehensive Inquiry into the Nature, Origin, and Fabrication of Life.* New York, NY: Columbia University Press.

[B160] Rosen R. (2012). *Anticipatory Systems: Philosophical, Mathematical, and Methodological Foundations*, 2nd Edn. Berlin, DE: Springer. 10.1007/978-1-4614-1269-4

[B161] Ruiz-Mirazo K. Moreno A. (2012). Autonomy in evolution: from minimal to complex life. *Synthese* 185 21–52. 10.1007/s11229-011-9874-z

[B162] Saborido C. Mossio M. Moreno A. (2011). Biological organization and cross-generation functions. *Br. J. Philos. Sci.* 62 583–606. 10.1093/bjps/axq034 32691291

[B163] Savage L. J. (1954). *The Foundations of Statistics.* New York, NY: John Wiley & Sons.

[B164] Schotanus P. Chrisley R. Clark A. Pritchard D. Schurger A. (2020). *Reflexivity and the Market Mind Hypothesis: Why George Soros is Not a Failed Philosopher (and What it Means for Economics, the Economy, and Investing) [SSRN Scholarly Paper 3939493].* Available online at: 10.2139/ssrn.3939493 (accessed December 16, 2020).

[B165] Seth A. (2021). *Being You: a New Science of Consciousness.* Dutton.

[B166] Shadlen M. N. Gold J. I. (2004). “The neurophysiology of decision-making as a window on cognition,” in *The Cognitive Neurosciences*, 3rd Edn, ed. Gazzaniga M. S. (Cambridge, MA: The MIT Press), 1229–1241.

[B167] Shapiro J. A. (2007). Bacteria are small but not stupid: cognition, natural genetic engineering and socio-bacteriology. *Stud. Hist. Philos. Sci. C Biol. Biomed. Sci.* 38 807–819. 10.1016/j.shpsc.2007.09.010 18053935

[B168] Shapiro L. Spaulding S. (2021). “Embodied cognition,” in *The Stanford Encyclopedia of Philosophy (Winter 2021)*, ed. Zalta E. N. (Stanford, CA: Metaphysics Research Lab, Stanford University).

[B169] Shields C. (2020). “Aristotle’s psychology,” in *The Stanford Encyclopedia of Philosophy (Winter 2020)*, ed. Zalta E. N. (Stanford, CA: Metaphysics Research Lab, Stanford University).

[B170] Simon H. A. (1956). Rational choice and the structure of the environment. *Psychol. Rev.* 63 129–138. 10.1037/h0042769 13310708

[B171] Simon H. A. (1957). *Models of Man: Social and Rational - Mathematical Essays on Rational Human Behavior in a Social Setting.* New York, NY: John Wiley & Sons.

[B172] Simon H. A. (1989). “The scientist as problem solver,” in *Complex information processing: The impact of Herbert A. Simon*, eds Klahr D. Kotovsky K. (Hillsdale, NJ: Lawrence Erlbaum Associates, Inc), 375–398.

[B173] Simon H. A. (1990). Invariants of human behavior. *Annu. Rev. Psychol.* 41 1–19. 10.1146/annurev.ps.41.020190.000245 18331187

[B174] Simon H. A. Newell A. (1958). Heuristic problem solving: the next advance in operations research. *Operations Res.* 6 1–10. 10.1287/opre.6.1.1 19642375

[B175] Sims M. (2021). A continuum of intentionality: linking the biogenic and anthropogenic approaches to cognition. *Biol. Philos.* 36:51. 10.1007/s10539-021-09827-w

[B176] Soto A. M. Longo G. Miquel P.-A. Montevil M. Mossio M. Perret N. (2016b). Toward a theory of organisms: three founding principles in search of a useful integration. *Prog. Biophys. Mol. Biol.* 122 77–82. 10.1016/j.pbiomolbio.2016.07.006 27498204 PMC5097676

[B177] Soto A. M. Longo G. Montévil M. Sonnenschein C. (2016a). The biological default state of cell proliferation with variation and motility, a fundamental principle for a theory of organisms. *Prog. Biophys. Mol. Biol.* 122 16–23. 10.1016/j.pbiomolbio.2016.06.006 27381480 PMC5659334

[B178] Sperber D. Wilson D. (1986). *Relevance: Communication and Cognition.* Oxford: Wiley–Blackwell.

[B179] Sperber D. Wilson D. (1996). Fodor’s frame problem and relevance theory. *Behav. Brain Sci.* 19 530–532. 10.1017/S0140525X00082030

[B180] Stanford P. K. (2010). *Exceeding Our Grasp: Science, History, and the Problem of Unconceived Alternatives.* Oxford: Oxford University Press.

[B181] Stoffregen T. A. (2003). Affordances as properties of the animal–environment system. *Ecol. Psychol.* 15 115–134. 10.1207/S15326969ECO1502_2

[B182] Sumers T. Hawkins R. Ho M. K. Griffiths T. L. (2022). Reconciling truthfulness and relevance via decision-theoretic utility. *PsyArXiv [Preprint]* 10.31234/osf.io/e9m3j 37589706

[B183] Sutton R. S. Barto A. G. (1998). *Reinforcement Learning: an Introduction*, 2nd Edn. Cambridge, MA: Bradford Books.

[B184] Szathmáry E. (2015). Toward major evolutionary transitions theory 2.0. *Proc. Natl. Acad. Sci. U.S.A.* 112 10104–10111. 10.1073/pnas.1421398112 25838283 PMC4547294

[B185] Thompson E. (2007). *Mind in Life: Biology, Phenomenology, and the Sciences of Mind.* Cambridge, MA: Belknap Press.

[B186] Tuckett D. Taffler R. (2008). Phantastic objects and the financial market’s sense of reality: a psychoanalytic contribution to the understanding of stock market instability. *Intl. J. Psychoanalysis* 89 389–412. 10.1111/j.1745-8315.2008.00040.x 18405290

[B187] Turing A. M. (1937). On computable numbers, with an application to the entscheidungsproblem. *Proc. London Math. Soc.* s2-42 230–265. 10.1112/plms/s2-42.1.230

[B188] Varela F. G. Maturana H. R. Uribe R. (1974). Autopoiesis: the organization of living systems, its characterization and a model. *BioSystems* 5 187–196. 10.1016/0303-2647(74)90031-8 4407425

[B189] Varela F. J. (1979). *Principles of Biological Autonomy.* New York, NY: North Holland.

[B190] Varela F. J. Thompson E. Rosch E. (1991). *The Embodied Mind: Cognitive Science and Human Experience.* The MIT Press: Cambridge, MA. 10.7551/mitpress/6730.001.0001

[B191] Vervaeke J. Ferraro L. (2013a). “Relevance, meaning and the cognitive science of wisdom,” in *The Scientific Study of Personal Wisdom*, eds Ferrari M. Weststrate N. M. (Dordrecht, NL: Springer Netherlands), 21–51. 10.1007/978-94-007-7987-7_2

[B192] Vervaeke J. Ferraro L. (2013b). “Relevance realization and the neurodynamics and neuroconnectivity of general intelligence,” in *SmartData*, eds Harvey I. Cavoukian A. Tomko G. Borrett D. Kwan H. Hatzinakos D. (New York, NY: Springer), 57–68. 10.1007/978-1-4614-6409-9_6

[B193] Vervaeke J. Mastropietro C. (2021a). Dialectic into dialogos and the pragmatics of no-thingness in a time of crisis. *Eidos* 5 58–77. 10.14394/eidos.jpc.2021.0017

[B194] Vervaeke J. Mastropietro C. (2021b). “Gnosis in the second person: responding to the meaning crisis in the socratic quest of authentic dialogue,” in *Metamodernity—Dispatches from a Time Between Worlds*, eds Rowson J. Layman P. (London: Perspectiva Press), 241–270.

[B195] Vervaeke J. Lillicrap T. P. Richards B. A. (2012). Relevance realization and the emerging framework in cognitive science. *J. Logic. Comput.* 22 79–99. 10.1093/logcom/exp067

[B196] Vervaeke J. Mastropietro C. Miscevic F. (2017). *Zombies in Western Culture: A Twenty-First Century Crisis.* Cambridge: Open Book Publishers. 10.11647/OBP.0113

[B197] von Uexküll J. (1909). *Umwelt und Innenwelt der Tiere.* Berlin, DE: Julius Springer.

[B198] Walsh D. (2012). Mechanism and purpose: a case for natural teleology. *Stud. Hist. Philos. Sci. C Biol. Biomed. Sci.* 43 173–181. 10.1016/j.shpsc.2011.05.016 22326086

[B199] Walsh D. (2015). *Organisms, Agency, and Evolution.* Cambridge: Cambridge University Press. 10.1017/CBO9781316402719

[B200] Walsh D. M. (2012). “Situated adaptationism,” in *The Environment: Philosophy, Science, and Ethics*, eds Kabasenche W. P. O’Rourke M. Slater M. H. (Cambridge, MA: The MIT Press), 89–116. 10.7551/mitpress/9780262017404.003.0006

[B201] Walsh D. M. (2013). “Mechanism, emergence, and miscibility: the autonomy of evo-devo,” in *Functions: Selection and mechanisms*, ed. Huneman P. (Dordrecht, NL: Springer Netherlands), 43–65. 10.1007/978-94-007-5304-4_3

[B202] Walsh D. M. (2014). “The affordance landscape: the spatial metaphors of evolution,” in *Entangled Life*, eds Barker G. Desjardins E. Pearce T. (Dordrecht, NL: Springer Netherlands), 213–236. 10.1007/978-94-007-7067-6_11

[B203] Walsh D. M. Rupik G. (2023). The agential perspective: countermapping the modern synthesis. *Evol. Dev.* 25 335–352. 10.1111/ede.12448 37317654

[B204] Weber A. Varela F. J. (2002). Life after Kant: natural purposes and the autopoietic foundations of biological individuality. *Phenomenol. Cogn. Sci.* 1 97–125. 10.1023/A:1020368120174

[B205] Weizenbaum J. (1976). *Computer Power and Human Reason: from Judgment to Calculation.* San Francisco, CA: W.H. Freeman.

[B206] West S. A. Fisher R. M. Gardner A. Kiers E. T. (2015). Major evolutionary transitions in individuality. *Proc. Natl. Acad. Sci. U S A.* 112 10112–10119. 10.1073/pnas.1421402112 25964342 PMC4547252

[B207] Wheeler M. (2012). “Naturalizing dasein and other (Alleged) Heresies,” in *Heidegger and Cognitive Science*, eds Kiverstein J. Wheeler M. (London: Palgrave Macmillan), 176–212. 10.1007/978-1-137-00610-3_6

[B208] Williams B. (1973). “The Makropulos case: reflections on the tedium of immortality,” in *Problems of the Self: Philosophical Papers 1956–1972*, ed. William B. (Cambridge: Cambridge University Press), 82–100. 10.1017/CBO9780511621253.008

[B209] Wilson D. (2017). “Relevance theory,” in *The Oxford Handbook of Pragmatics*, ed. Huang Y. (Oxford: Oxford University Press), 79–100. 10.1093/oxfordhb/9780199697960.013.25

[B210] Wilson D. Sperber D. (2012). *Meaning and Relevance.* Cambridge: Cambridge University Press. 10.1017/CBO9781139028370

[B211] Wilson R. C. Niv Y. (2012). Inferring relevance in a changing world. *Front. Hum. Neurosci.* 5:189. 10.3389/fnhum.2011.00189 22291631 PMC3264902

[B212] Wimsatt W. C. (2007). *Re-Engineering Philosophy for Limited Beings: Piecewise Approximations to Reality.* Cambridge, MA: Harvard University Press. 10.2307/j.ctv1pncnrh

[B213] Wrathall M. Malpas J. (2000). *Heidegger, Coping, and Cognitive Science: Essays in Honor of Hubert L. Dreyfus*, Vol. 2. Cambridge, MA: The MIT Press.

[B214] Zach R. (2023). “Hilbert’s program,” in *The Stanford Encyclopedia of Philosophy* (Spring 2023), eds Zalta E. N. Nodelman U. (Stanford, CA: Metaphysics Research Lab, Stanford University).

[B215] Zebrowski R. L. McGraw E. B. (2021). Autonomy and openness in human and machine systems: participatory sense-making and artificial minds. *J. Artif. Intell. Conscious.* 8 303–323. 10.1142/S2705078521500181

